# RSV temporally reprograms apoptosis and pyroptosis to balance immune evasion and replication

**DOI:** 10.1126/sciadv.adz2496

**Published:** 2026-01-23

**Authors:** Cong Liu, Haiwu Zhou, Jian Li, Yang Meng, Jingyu Wang, Mingbin He, Weiwei Wang, Zhifei Li, Yali Qin, Mingzhou Chen

**Affiliations:** ^1^ School of Life Sciences, Hubei University, Wuhan, China.; ^2^ State Key Laboratory of Virology and Biosafety, Hubei Provincial Research Center for Basic Biological Sciences, College of Life Sciences, Wuhan University, Wuhan, China.

## Abstract

Virus-induced inflammation and programmed cell death (PCD) are critical antiviral defenses, prompting viruses like respiratory syncytial virus (RSV) to develop PCD regulation mechanisms. Here, we demonstrate that RSV orchestrates the temporal and sequential regulation of distinct PCD pathways in human macrophages to optimize replication and dissemination. During early stages of infection, RSV activates the PI3K-Akt pathway to induce cFLIP expression, effectively suppressing TNF-driven extrinsic apoptosis. Simultaneously, viral degradation of ZDHHC9 prevents GSDMD-mediated pyroptosis downstream of NLRP3 activation, thereby sustaining an intracellular environment permissive to viral propagation. In contrast, following the completion of replication, RSV subverts caspase-1 signaling to trigger the intrinsic apoptotic cascade via the Casp-1–BID–APAF1–Casp-9 axis, and subsequently promotes GSDME-mediated secondary pyroptosis. This late-stage PCD reprogramming enables synchronized release of virions and pro-inflammatory cytokines, exacerbating pulmonary pathology. These findings delineate a temporally resolved strategy by which RSV balances early immune evasion with subsequent viral dissemination and immunopathology, and identify discrete stage-specific molecular targets for therapeutic intervention in RSV-induced lung disease.

## Teaser

RSV orchestrates cell death and IL-1β release via spatially distinct, linked death signaling axes for immune evasion and spread.

## INTRODUCTION

Respiratory syncytial virus (RSV), an enveloped, nonsegmented negative-sense RNA virus of the *Orthopneumovirus* genus (*Pneumoviridae* and *Mononegavirales*), is a major cause of respiratory infection (*1*, *2*). While RSV typically induces self-limited, mild illness in immunocompetent individuals (*3*, *4*), it poses substantial risks to infants, the elderly, and immunocompromised populations, manifesting as severe bronchiolitis or pneumonia (*4*, *5*). In rare acute cases, neurological complications and mortality may ensue (*1*, *5*). Current global estimates indicate that RSV is responsible for approximately 33 million lower respiratory tract infections in children under five each year, accounting for over 3.6 million hospitalizations and more than 110,000 deaths (*6*). This substantial disease burden persists, in large part, due to the virus’s sophisticated immune evasion strategies, which have thwarted effective vaccine development (*7*, *8*). Elucidating the host cell death and inflammatory signaling networks manipulated by RSV is therefore essential for rational design of targeted interventions.

Programmed cell death (PCD), encompassing apoptosis, necroptosis, pyroptosis, and ferroptosis, represents an evolutionarily conserved defense strategy. These forms of PCD not only maintain tissue homeostasis by removing compromised or malignant cells but also restrict pathogen propagation (*9*–*11*). Apoptosis, divided into intrinsic and extrinsic pathways, is orchestrated by a tightly regulated cascade of caspases (*12*). Intrinsic apoptosis is triggered by internal stress signals, such as DNA damage, resulting in mitochondrial outer membrane permeabilization, cytochrome c release, apoptosome assembly, and activation of caspase-9 and its downstream executioner caspases, caspase-3 and caspase-7. Extrinsic apoptosis proceeds via engagement of death receptors, such as tumor necrosis factor receptor (TNFR) or Fas, leading to formation of the death-inducing signaling complex (DISC) and subsequent activation of caspases-8 or caspases-10 that converge on the executioner caspases. Activated executioner caspases cleave key substrates such as poly(adenosine diphosphate–ribose) polymerase (PARP) and Lamin A/C, triggering cellular shrinkage, cytoskeletal disintegration, chromatin condensation, and nuclear fragmentation (*9*, *11*). By rapidly eliminating infected cells, apoptosis minimizes inflammation and preserves tissue integrity, thereby effectively limiting viral spread (*13*).

In contrast, pyroptosis is a highly inflammatory mode of PCD, mediated by the gasdermin family. Proteolytic cleavage of gasdermin proteins releases their N-terminal domains, which oligomerize to form pores in the plasma membrane, resulting in lytic cell death and release of proinflammatory contents. Gasdermin D (GSDMD) and Gasdermin E (GSDME) are cleaved by inflammatory caspases (caspase-1/-4/-5/-8/-11) or apoptotic caspase-3, respectively, to initiate pyroptosis (*14*). Canonical pyroptosis relies on inflammasome activation: multiprotein complexes assembled by cytosolic sensors [e.g., NLRP3, NACHT, leucine-rich repeat, and pyrin domain (PYD)–containing protein 3, or AIM2, absent in melanoma-2], the adaptor ASC (apoptosis-associated speck-like protein), and caspase-1 (*15*). Upon recognition of pathogen- or damage-associated molecular patterns (PAMPs/DAMPs), inflammasomes oligomerize, leading to caspase-1 activation, which drives both GSDMD cleavage and processing of the proinflammatory cytokines pro–interleukin-1β (pro-IL-1β) and pro-IL-18. Pores formed by N-terminal GSDMD (N-GSDMD) facilitate the extracellular release of mature cytokines, amplifying immune activation (*16*). Virus-induced pyroptosis, therefore, exerts double-edged effects: enhancing host defense by promoting cytokine secretion and immune recruitment, but also contributing to immunopathology when excessive (*10*, *15*).

Extensive cross-talk exists among PCD pathways. For example, caspase-1, activated by inflammasomes, can trigger intrinsic apoptosis via BH3 interacting domain death agonist (BID) cleavage in the absence of functional GSDMD (*17*); conversely, ASC can scaffold caspase-8 activation, initiating extrinsic apoptosis when caspase-1 is lacking (*18*, *19*). Caspase-8 can also cleave GSDMD to induce pyroptosis under inhibition of transforming growth factor β-activated kinase 1 (TAK1) (*20*, *21*). Upon caspase-8 inhibition or deficiency, the ubiquitination of receptor-interacting protein kinase 1 (RIPK1) within the DISC is dissociated. Through RHIM-RHIM interaction, RIPK1 autophosphorylates and trans-phosphorylates RIPK3. RIPK3 then recruits its executioner substrate, mixed lineage kinase domain-like pseudokinase (MLKL), leading to the assembly of the RIPK1-RIPK3-MLKL necrosome and the induction of necroptosis (*9*, *11*, *12*). Furthermore, in GSDME-expressing cells, caspase-3, activated during apoptosis, cleaves GSDME and drives secondary pyroptosis, blurring boundaries between death modalities (*22*, *23*). Caspase-3 can also cleave GSDMD into an inactive form, attenuating GSDMD-dependent pyroptosis (*24*). These findings illustrate the intricate regulation and interplay of PCD pathways in antiviral defense and disease.

Upon pathogen encounter, macrophages secrete cytokines such as IL-6, IL-8, TNF, and C-C motif chemokine ligands (CCLs) to recruit monocytes to sites of infection, an essential process for effective host defense and maintenance of immune homeostasis (*25*). Although premature cell death can limit viral replication, many viruses have evolved strategies to suppress cell death and immune activation to enable their persistence (*26*). Epidemiological data indicate that approximately 30% of RSV-infected adults, as well as subsets of infants and elderly individuals, display minimal or mild upper respiratory symptoms, chiefly nasal congestion and low-grade fever (*27*, *28*). However, prolonged infection in some patients can progress rapidly to severe lower respiratory diseases, including bronchitis and pneumonia (*5*, *29*). The immunopathology characteristic of RSV-associated pneumonia arises from excessive pro-inflammatory responses, driven by dysregulated cytokine production from infected immune cells (*8*, *29*). Lytic cell death further amplifies tissue injury via the release of DAMPs (*30*). Notably, during the initial 24 hours of infection, RSV does not provoke substantial inflammatory signaling or cell death (*8*, *25*). Viral mRNA and proteins become detectable as early as 4 to 8 hours postinfection and the generation of progeny virions is observed from 10 hours postinfection onward (*31*, *32*). These temporal dynamics raise unresolved questions: Why do immune cells fail to mount a swift antiviral response to restrict RSV at the outset, and how does the virus manipulate cellular fate to support its replication? Despite prior reports describing RSV-induced inflammatory and cell death pathways (*25*, *33*), the molecular basis for the coordinated regulation of diverse PCD by RSV remains poorly characterized.

In this study, we delineate the molecular framework through which RSV orchestrates sequential PCD pathways to maximize replication and dissemination. During early infection, RSV engages a dual strategy: Suppression of extrinsic apoptosis via phosphoinositide 3-kinase (PI3K)–protein kinase B (Akt)–dependent up-regulation of cellular FLICE (FADD-like IL-1β–converting enzyme) inhibitory protein (cFLIP), and inhibition of N-GSDMD–driven pyroptosis through targeted degradation of zinc finger Asp-His-His-Cys-9 (ZDHHC9). These mechanisms collectively subvert host antiviral defenses and establish an intracellular environment conducive to robust viral multiplication. Upon completion of the replication cycle, RSV redirects signaling to initiate a Casp-1–BID–apoptotic protease activating factor-1 (APAF1)–Casp-9 axis, triggering intrinsic apoptosis, followed by N-terminal GSDME (N-GSDME)–mediated secondary pyroptosis. This temporally regulated cell death switch ensures synchronized release of mature virions and inflammatory mediators, promoting viral propagation while exacerbating lung inflammation. Together, these findings define the central role of spatiotemporal PCD modulation in RSV pathogenesis and provide critical mechanistic insights for the rational development of targeted antiviral therapies.

## RESULTS

### RSV infection elicits distinct and dysregulated inflammatory responses in macrophages

Despite inducing robust secretion of proinflammatory mediators such as IL-6, IL-8, and TNF, RSV infection does not elicit a marked increase in IL-1β levels in bronchoalveolar lavage fluid, nasopharyngeal secretions, or systemic circulation during the early phase of infection (*25*, *34*). To explore the cytopathic effects (CPEs) of RSV and the production and release of IL-1β, we infected a panel of immune cells (THP-1 macrophages) and nonimmune cells (HeLa, A549, HEp-2, and BEAS-2B) with RSV. The results showed that RSV infection triggered cell fusion and differentially up-regulated *IL-1*β mRNA in nonimmune cells by 24 hours postinfection, yet it failed to elicit IL-1β secretion (fig. S1, A to D). In contrast, in phorbol 12-myristate 13-acetate (PMA)–differentiated THP-1 macrophages, a human monocytic model expressing canonical inflammasome and PCD components, while RSV rapidly elevated *IL-1*β mRNA levels, substantial IL-1β release was detected only at late time points (24 hours postinfection) (fig. S1, C and D).

**Fig. 1.**
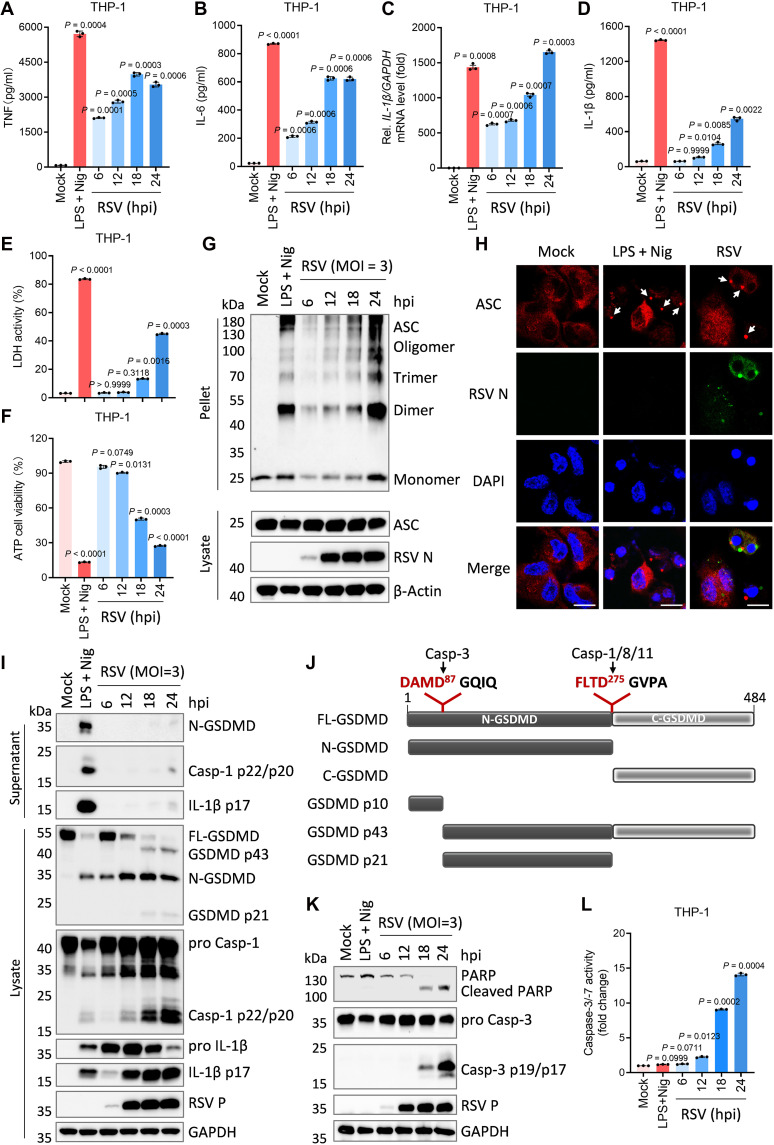
RSV-infected macrophages exhibit abnormal and complex inflammatory status. (**A** to **G**) PMA-differentiated THP-1 macrophages were infected with RSV at an MOI of 3.0 for indicated times. Supernatants were collected and subjected to measurements of TNF (A), IL-6 (B), IL-1β (D), and LDH release (E). The intracellular mRNA levels of *IL-1*β were measured by RT-qPCR, and the levels of *IL-1*β mRNA in mock cells were defined as onefold (D). The percentage of viable cells was determined through cell viability (ATP Glo) evaluation (F). ASC oligomerization was analyzed by immunoblots (G). (**H**) Representative immunofluorescence images of endogenous ASC specks (arrows) in PMA-differentiated THP-1 macrophages infected by RSV (MOI = 3.0) for 24 hours. Scale bars, 20 μm. (**I**) Immunoblots of the indicated proteins in the supernatants and lysates of PMA-differentiated THP-1 macrophages infected with RSV (MOI = 3.0) for indicated times. (**J**) Schematic illustration of human GSDMD with the functional domains and protease cleavage sites. (**K** and **L**) Immunoblot of the indicated proteins (K) and caspase-3/-7 activity (L) in the lysates of PMA-differentiated THP-1 macrophages infected with RSV (MOI = 3.0) for indicated times. As a positive control of pyroptosis, cells were treated with LPS (1 μg/ml) for 4 hours plus Nig (2.5 μM) (LPS + Nig) for 2 hours. Graphs show mean ± SD (*n* = 3 biologically independent experiments). Statistical significance was determined by one-way ANOVA in [(A) to (F) and (L)]. Casp, caspase; FL-GSDMD, full-length GSDMD; N-GSDMD, N-terminal GSDMD; C-GSDMD, C-terminal GSDMD; hpi, hours postinfection.

To investigate the inflammatory and immunomodulatory cascades triggered by RSV, we infected THP-1 cells with a high dose of the virus [multiplicity of infection (MOI) = 3.0]. Time-resolved profiling of cytokine release and cell death was conducted in supernatants from PMA-differentiated THP-1 macrophages following RSV infection. Lipopolysaccharide (LPS) with nigericin (Nig; an NLRP3 activator) served as a positive control. Lactate dehydrogenase (LDH) release and adenosine triphosphate (ATP) depletion were quantified as indicators of plasma membrane integrity and cell viability, respectively. RSV infection elicited rapid and substantial TNF and IL-6 secretion within 12 hours (Fig. 1, A and B), concurrently with a strong up-regulation of *IL-1*β mRNA levels (Fig. 1C), whereas the release of mature IL-1β was delayed and modest (Fig. 1D). Markers of lytic death (LDH/ATP) became detectable only after 18 hours postinfection (Fig. 1, E and F).

The production of TNF and IL-6 is driven by Toll-like receptor (TLR) signaling (*35*) independently of cell membrane rupture associated with cell death. In contrast, IL-1β maturation and LDH release are dependent on caspase-1 cleavage after inflammasome activation and subsequent cell membrane disruption (*15*). The critical involvement of caspases in this RSV-triggered death pathway was collectively demonstrated by inhibition with Z-VAD-FMK (pan-caspase inhibitor), Nec-1s (necroptosis inhibitor), and ferrostatin-1 (Fer-1; ferroptosis inhibitor) (fig. S1, A to D). Pharmacological inhibition with MCC950 (NLRP3 inhibitor) or VX765 (caspase-1 inhibitor), as well as CRISPR-mediated knockout of inflammasome components, confirmed that RSV-induced processing of pro-IL-1β is strictly dependent on NLRP3 inflammasome (fig. S1, E to H).

Release of mature IL-1β and LDH is classically mediated by pyroptosis following caspase-1–dependent GSDMD cleavage. To address whether the delayed cytokine and LDH release reflects incomplete inflammasome activation, we analyzed inflammasome component expression and cleavage, alongside verification of RSV replication by nucleocapsid (N) and phosphoprotein (P) expression. Western blot and confocal imaging demonstrated that RSV rapidly induces ASC oligomerization and speck assembly upon infection (Fig. 1, G and H). Robust caspase-1 activation and proteolytic maturation of pro-IL-1β and GSDMD were observed, indicating prompt NLRP3 inflammasome activation (Fig. 1I). However, in contrast to LPS/Nig, RSV elicited minimal release of active caspase-1 p22/p20, mature IL-1β p17, and N-GSDMD (Fig. 1I). Notably, two aberrant GSDMD cleavage products (p43 and p21) emerged at 18 hours postinfection (Fig. 1I), consistent with noncanonical processing by the apoptotic executioner caspase, caspase-3. As schematized in Fig. 1J, caspase-3 cleaves GSDMD at D87 to generate the p43 fragment (from full-length GSDMD) and the p21 fragment (from N-GSDMD). Immunoblot and caspase activity analysis confirmed robust caspase-3 activation and PARP cleavage (classical apoptosis markers) by 18 hours postinfection (Fig. 1, K and L).

Together, these data reveal that RSV-infected macrophages mount an early proinflammatory response, characterized by TNF and IL-6 production, while also activating the NLRP3 inflammasome. Despite upstream caspase-1 activation and GSDMD cleavage, efficient IL-1β and LDH release is contingent upon late-stage apoptosis, implicating apoptotic rather than canonical pyroptotic mechanisms in RSV-driven immunopathology.

### GSDME, rather than GSDMD, mediates IL-1β secretion downstream of NLRP3 inflammasome

**Fig. 2.**
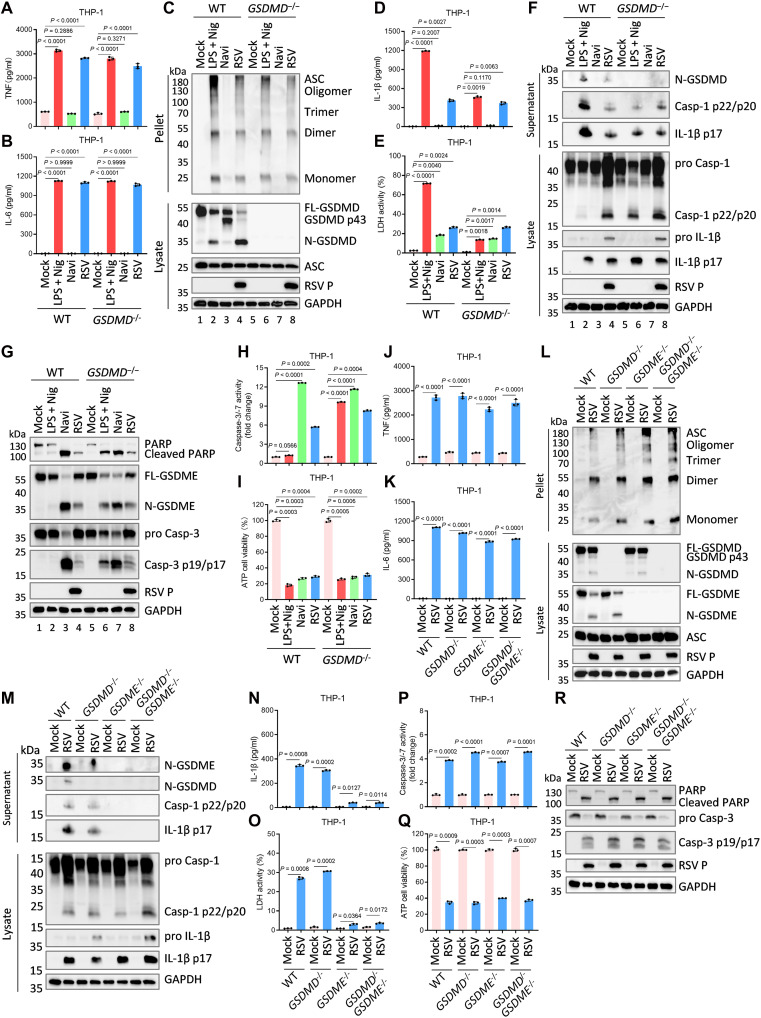
RSV-induced IL-1β secretion downstream of NLRP3 inflammasome depends on GSDME, not GSDMD. (**A** to **I**) PMA-differentiated WT or *GSDMD* knockout (*GSDMD*^−/−^) THP-1 macrophages were infected with RSV (MOI = 3.0) for 24 hours or treated with positive controls of pyroptosis [LPS (1 μg/ml; 4 hours) plus Nig (2.5 μM, 2 hours) (LPS + Nig)] or apoptosis [navitoclax (Navi) (50 μM, 5 hours)]. Supernatants were collected and subjected to measurements of TNF (A), IL-6 (B), IL-1β (D), and LDH release (E). Cell supernatant, pellets, and lysates were analyzed by immunoblots (C, F, and G), caspase-3/-7 activity assay (H), and cell viability (ATP Glo) evaluation (I). (**J** to **R**) PMA-differentiated WT, *GSDMD*^−/−^, *GSDME*^−/−^, or *GSDMD*^−/−^*GSDME*^−/−^ THP-1 macrophages were infected with RSV (MOI = 3.0) for 24 hours. Supernatants were collected and subjected to measurements of TNF (J), IL-6 (K), IL-1β (N), and LDH release (O). Cell supernatant, pellets, and lysates were analyzed by immunoblots (L, M, and R), caspase-3/-7 activity assay (P), and cell viability (ATP Glo) evaluation (Q). Graphs show mean ± SD (*n* = 3 biologically independent experiments). Statistical significance was determined by two-way ANOVA in [(A), (B), (D), (E), (H) to (K), and (N) to (Q)]. Casp, caspase; FL-GSDMD, full-length GSDMD; N-GSDMD, N-terminal GSDMD; FL-GSDME, full-length GSDME; N-GSDME, N-terminal GSDME.

To determine the effector mediator of RSV-induced pyroptosis and IL-1β maturation, we generated *GSDMD*-deficient (*GSDMD*^−/−^) THP-1 cells. Deletion of *GSDMD* had no impact on TNF and IL-6 release in response to LPS plus Nig or RSV infection, nor did it impair NLRP3 inflammasome activation, as indicated by ASC oligomerization (Fig. 2, A to C). Consistently, RSV-infected *GSDMD*^−/−^ cells exhibited levels of pyroptosis and mature IL-1β release comparable to wild-type (WT) controls (Fig. 2, D to F, lanes 4 and 8). Notably, *GSDMD* ablation did not fully suppress LPS plus Nig-induced pyroptosis and IL-1β release (Fig. 2, D to F, lanes 2 and 6), supporting the established mechanism wherein, in the absence of *GSDMD*, inflammasome activation prompts caspase-1–dependent apoptosis, which can subsequently transition to GSDME-mediated pyroptosis (*17*, *23*). Accordingly, we detected caspase-3 activation and GSDME cleavage in *GSDMD*^−/−^ cells under these conditions (Fig. 2, G and H, lane 6). Similarly, RSV-induced caspase-3 activation, GSDME cleavage, and cell death did not differ between WT and *GSDMD*^−/−^ cells (Fig. 2, G to I, lanes 4 and 8). Apoptosis induction by Navi (navitoclax) also promoted caspase-3 and GSDME cleavage, yet did not generate substantial LDH release within the tested timeframe (Fig. 2G, lanes 3 and 7), which is similar to *GSDMD*^−/−^ cells treated with LPS plus Nig, the release of LDH in the cell supernatant were inconspicuous (Fig. 2D), likely reflecting limited progression of apoptosis to GSDME-mediated secondary pyroptosis (*36*). These results suggest that RSV-induced pyroptosis and mature IL-1β release may depend on GSDME in the apoptotic cascade, rather than GSDMD.

To resolve the definitive executioner of RSV-induced lytic death, we established *GSDME*^−/−^ and *GSDMD*^−/−^*GSDME*^−/−^ THP-1 cell lines. Deletion of either *GSDMD* or *GSDME* did not alter RSV-stimulated TNF and IL-6 secretion or NLRP3 inflammasome activation (Fig. 2, J to M). Consistent with prior results, RSV-infected *GSDMD*^−/−^ cells exhibited unimpaired pyroptosis and IL-1β release, concomitant with robust N-GSDME production (Fig. 2, M to O). In notable contrast, *GSDME* deficiency nearly eliminated RSV-induced pyroptosis and mature IL-1β release, despite retention of caspase-3 activation and apoptosis (Fig. 2, L to R). Collectively, these results establish that RSV activates the NLRP3 inflammasome and promotes GSDMD cleavage, but RSV-induced pyroptosis and IL-1β release are executed primarily through caspase-3–dependent GSDME activation following apoptosis.

### RSV induces GSDME-mediated secondary pyroptosis via the Casp-1–BID–APAF1–Casp-9 axis

**Fig. 3.**
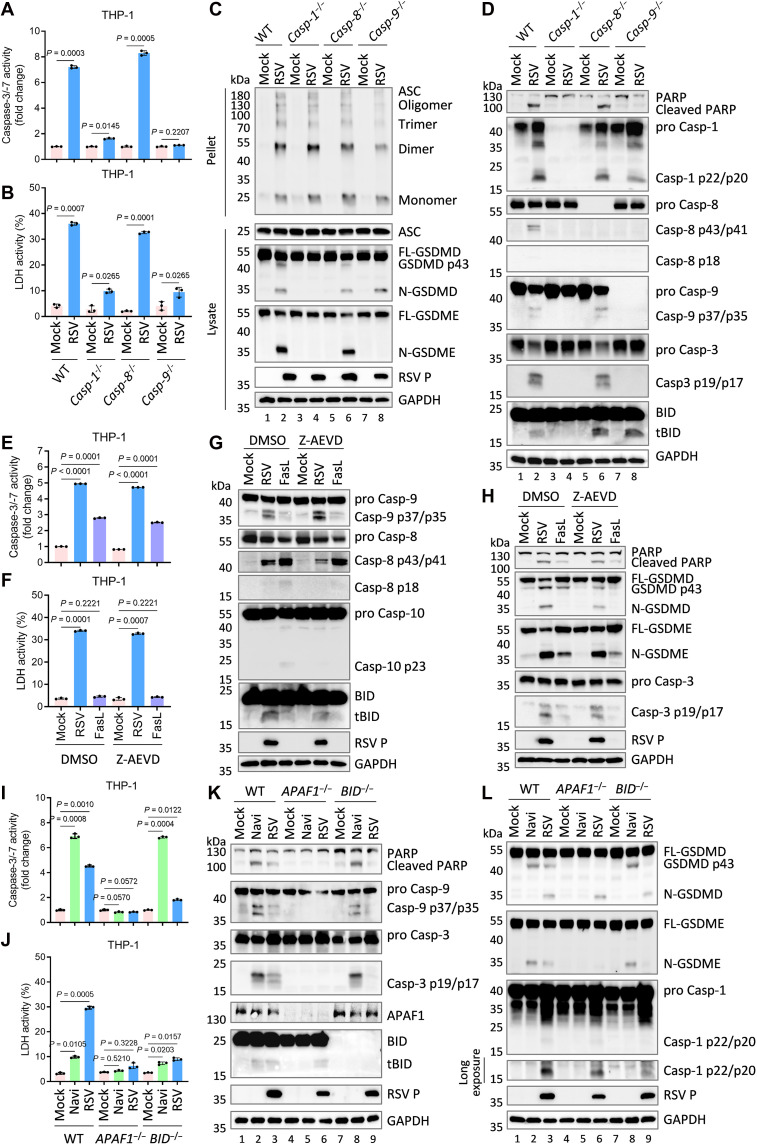
RSV drives intrinsic apoptosis and GSDME-dependent pyroptosis via the Casp-1–BID–APAF1–Casp-9 axis. (**A** to **D**) PMA-differentiated WT, *Casp-1*^−/−^, *Casp-8*^−/−^, or *Casp-9*^−/−^ THP-1 macrophages were infected with RSV (MOI = 3.0) for 24 hours. Cell lysates and pellets were analyzed by caspase-3/-7 activity assay (A) and immunoblots (C and D). Supernatants were collected and subjected to measurements of LDH release (B). (**E** to **H**) PMA-differentiated WT THP-1 macrophages were pretreated with dimethyl sulfoxide (DMSO) or caspase-10 inhibitor Z-AEVD-FMK (30 μM) for 1 hour, and then infected with RSV (MOI = 3.0) for 24 hours or stimulated with FasL (200 ng/ml) for 6 hours. Cell lysates and pellets were analyzed by caspase-3/-7 activity assay (E) and immunoblots (G and H). Supernatants were collected and subjected to measurements of LDH release (F). (**I** to **L**) PMA-differentiated WT, *APAF1*^−/−^, or *BID*^−/−^ THP-1 macrophages were infected with RSV (MOI = 3.0) for 24 hours. Cell lysates were analyzed by caspase-3/-7 activity assay (I) and immunoblots (K and L). Supernatants were collected and subjected to measurements of LDH release (J). Graphs show mean ± SD (*n* = 3 biologically independent experiments). Statistical significance was determined by two-way ANOVA in [(A), (B), (E), (F), (I), and (J)]. Casp, caspase; FL-GSDMD, full-length GSDMD; N-GSDMD, N-terminal GSDMD; FL-GSDME, full-length GSDME; N-GSDME, N-terminal GSDME.

GSDME is cleaved by caspase-3, activated downstream of either caspase-8 (extrinsic) or caspase-9 (intrinsic) apoptotic pathways (*11*). To elucidate the pathway engaged by RSV and its link to NLRP3 inflammasome activation, RSV infection was performed in WT, *caspase-1*–deficient (*Casp-1*^−/−^), *caspase-8*–deficient (*Casp-8*^−/−^), and *caspase-9*–deficient (*Casp-9*^−/−^) THP-1 macrophages. Loss of *caspase-9* completely abrogated RSV-induced apoptosis, while *caspase-8* deficiency had no effect, indicating that RSV specifically activates intrinsic, not extrinsic, apoptosis (Fig. 3, A to D, lanes 5 to 8). Notably, *caspase-9* deficiency permitted continued inflammasome activation and GSDMD cleavage, but blocked pyroptosis due to lack of caspase-3–GSDME signaling (Fig. 3, A to D, lanes 7 to 8). Notably, *caspase-1* deficiency markedly reduced both apoptosis and pyroptosis, despite preserved ASC oligomerization, revealing a central role for caspase-1 in triggering the intrinsic apoptotic pathway during RSV infection (Fig. 3, A to D, lanes 3 to 4).

To exclude CRISPR off-target effects, two *Casp-9*^−/−^ THP-1 clonal lines were compared with WT controls. Consistent with the cell death specificity, *Casp-9*^−/−^ abolished intrinsic apoptosis in response to Navi, but had no impact on pyroptosis or IL-1β release induced by LPS plus Nig (fig. S3, A to D). In contrast, RSV-infected *Casp-9*^−/−^ cells maintained inflammasome activation (caspase-1/GSDMD processing) but failed to execute pyroptosis and secrete inflammatory mediators (IL-1β p17, caspase-1 p22/p20, and N-GSDMD), supporting a post-inflammasome requirement for caspase-9 in GSDME-dependent pyroptotic execution (fig. S3, A, C, and D, lanes 4, 8, and 12). Thus, RSV-induced intrinsic apoptosis precedes and directs GSDME-mediated secondary pyroptosis, together with caspase-1 activation within the NLRP3 inflammasome.

To determine whether RSV induces necroptosis to release LDH following *caspase-8* knockout, we examined the activation of RIPK3 and MLKL in infected WT and *Casp-8*^−/−^ THP-1 cells. The results demonstrated that TNF plus SM-164 (an IAP antagonist) induced apoptosis in WT THP-1 cells, whereas in *Casp*-8^−/−^ cells, it triggered RIPK3/MLKL phosphorylation, resulting in necroptosis and LDH release (fig. S3, E to H). In contrast, neither RSV infection nor TNF plus 5z-7-oxozeaenol (5z7) treatment led to the phosphorylation of RIPK3 and MLKL in either WT or *Casp-8*^−/−^ THP-1 cells (Fig. 3, G and H).

Previous work shows that, in the absence of *GSDMD*, caspase-1 cleaves BID, producing truncated BID (tBID), which translocates to mitochondria and induces cytochrome c (Cyto-C) release (*17*, *37*). APAF1 then scaffolds apoptosome formation, recruiting and activating caspase-9 to trigger intrinsic apoptosis (*17*). We detected the cleavage of BID in both *Casp-8*^−/−^ and *Casp-9*^−/−^ THP-1 cells, whereas genetic ablation of *caspase-1* completely eliminated BID cleavage (Fig. 3D). Although studies indicate that caspase-10, similar to caspase-8, can cleave BID to initiate a caspase-9–dependent apoptotic cascade (*38*), our data rule out its role in RSV-induced cell death (Fig. 3, E to H). While RSV infection triggered BID cleavage, it did not activate caspase-10 (Fig. 3G). Furthermore, the specific caspase-10 inhibitor failed to attenuate the BID cleavage and did not affect the activation of caspase-3 or LDH release induced by RSV (Fig. 3, E to H).

To test whether RSV uses a Casp-1–BID–APAF1–Casp-9 axis, we generated *BID*^−/−^ and *APAF1*^−/−^ THP-1 cells. As expected, neither Navi treatment nor RSV triggered apoptosis in *APAF1*^−/−^ cells (Fig. 3, I and K, lanes 4 to 6). Although RSV still initiated caspase-1 and GSDMD processing in *APAF1*^−/−^ cells, pyroptosis was abrogated owing to impaired GSDME cleavage (Fig. 3, J and L, lane 6). *BID* deficiency blocked both RSV-induced apoptosis and pyroptosis, but did not affect Navi-induced apoptosis (Fig. 3, I to L, lane 9). In WT cells, both Navi and RSV promoted BID cleavage (Fig. 3K, lanes 2 and 3); however, in *APAF1*^−/−^ cells, only RSV, but not Navi, induced BID cleavage (Fig. 3K, lanes 5 and 6), consistent with caspase-1–mediated versus caspase-9–mediated processing, respectively (*37*, *39*). Collectively, these findings demonstrate that RSV infection drives intrinsic apoptosis and subsequent GSDME-mediated secondary pyroptosis through a Casp-1–BID–APAF1–Casp-9 signaling axis.

### RSV impairs inflammasome signaling through GSDMD inactivation

**Fig. 4.**
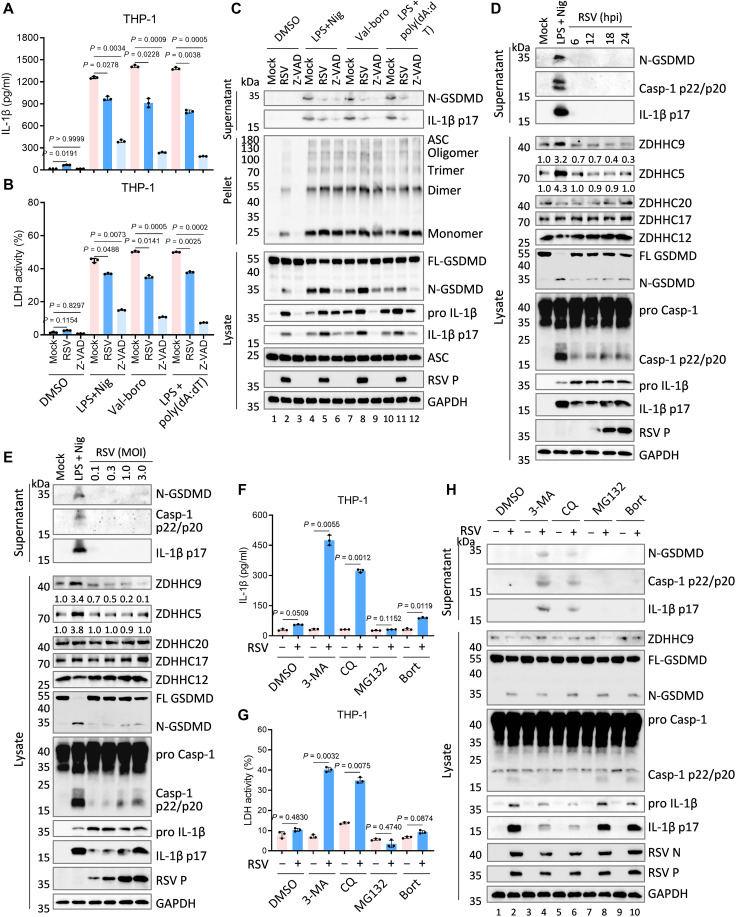
RSV impairs N-GSDMD–mediated pyroptosis by down-regulating the expression of ZDHHC9. (**A** to **C**) PMA-differentiated *Casp-9*^−/−^ THP-1 macrophages were infected with RSV (MOI = 3.0) for 6 to 8 hours or pretreated with pan-caspase inhibitor Z-VAD-FMK (30 μM) for 1 hour, and then stimulated with LPS (1 μg/ml) for 4 hours plus nigericin (Nig; 2.5 μM) for 1 hour, Val-boroPro mesylate (Val-boro; 20 μM) for 6 hours, or LPS (1 μg/ml) plus poly(dA:dT) (2 μg/ml) for 6 hours. Cell supernatants were collected and subjected to measurements of IL-1β secretion (A) and LDH release (B). Cell supernatant, pellets, and lysates were analyzed by immunoblots (C). (**D** and **E**) PMA-differentiated *Casp-9*^−/−^ THP-1 macrophages were infected with RSV at MOI = 3.0 for indicated times (D) or at different MOI for 12 hours (E), with LPS (1 μg/ml) for 4 hours plus Nig (2.5 μM) for 2 hour as the positive control of pyroptosis. Cell supernatants and lysates were analyzed by immunoblots. Quantified band intensities are shown for ZDHHC5 and ZDHHC9. (**F** and **H**) PMA-differentiated *Casp-9*^−/−^ THP-1 macrophages were infected with RSV (MOI = 3.0) for 12 hours in the presence/absence of DMSO, 3-methyladenine (3-MA; 1 mM), chloroquine (CQ; 1 μM), MG132 (1 μM), or bortezomib (Bort; 100 nM). Cell supernatants were collected and subjected to measurements of IL-1β secretion (F) or LDH release (**G**). Cell supernatant, pellets, and lysates were analyzed by immunoblots (H). Graphs show mean ± SD (*n* = 3 biologically independent experiments). Statistical significance was determined by two-way ANOVA in [(A), (B), (F), and (G)]. Casp, caspase; FL-GSDMD, full-length GSDMD; N-GSDMD, N-terminal GSDMD; hpi, hours postinfection.

Given that RSV-infected *Casp-9*^−/−^ cells exhibit caspase-1–dependent GSDMD cleavage yet fail to undergo pyroptosis (fig. S3), we hypothesized that RSV suppresses the pyroptotic activity of N-GSDMD. To test this, we challenged RSV-infected *Casp9*^−/−^ cells with distinct inflammasome activators, LPS plus Nig (NLRP3), Val-boroPro mesylate (Val-boro) (NLRP1), or LPS plus poly(dA:dT) (AIM2), and assessed inflammasome activation and GSDMD-dependent pyroptosis. The pan-caspase inhibitor Z-VAD-FMK robustly inhibited GSDMD and pro-IL-1β cleavage, as well as pyroptosis, across all conditions (Fig. 4, A to C, lanes 3, 6, 9, and 12). Notably, although RSV further increased GSDMD and pro-IL-1β cleavage upon inflammasome activation, it paradoxically suppressed N-GSDMD–mediated pyroptosis and IL-1β release (Fig. 4, A to C, lanes 2, 5, 8, and 11).

In THP-1 cells, the pore-forming activity of N-GSDMD requires palmitoylation by the acyltransferases ZDHHC5 and ZDHHC9 (*40*, *41*). We therefore examined whether RSV impairs the expression or function of ZDHHC proteins to inhibit GSDMD-dependent pyroptosis and IL-1β release. *Casp9^−/−^* THP-1 cells excluded confounding effects from apoptosis and GSDME-mediated pyroptosis. As expected, LPS plus Nig markedly increased the protein levels of both ZDHHC5 and ZDHHC9 and induced robust pyroptosis and IL-1β release (Fig. 4, D and E). In contrast, RSV selectively suppressed ZDHHC9 expression without affecting ZDHHC5, thus blocking GSDMD-mediated pyroptosis and IL-1β release (Fig. 4, D and E). Reverse transcriptase quantitative polymerase chain reaction (RT-qPCR) analysis confirmed robust mRNA induction of both enzymes by LPS plus Nig, but not by RSV, indicating post-transcriptional down-regulation of *ZDHHC9* (fig. S4).

Since RSV is known to exploit the autophagy-lysosomal pathway for targeted degradation of host antiviral factors (*42*, *43*), we tested whether this mechanism depletes ZDHHC9. Inhibitors of autophagy-lysosomal degradation: 3-methyladenine (3-MA) and chloroquine (CQ), but not proteasome inhibitors: MG132 and bortezomib (Bort), restored ZDHHC9 protein levels in RSV-infected cells, thereby rescuing N-GSDMD–dependent pyroptosis and IL-1β secretion (Fig. 4, F to H). Notably, autophagy inhibition also reduced RSV N and P protein expression, suggesting concomitant suppression of viral replication (Fig. 4H, lanes 4 and 6).

Together, these results demonstrate that RSV inhibits N-GSDMD–mediated pyroptosis by selectively degrading ZDHHC9 via the autophagy-lysosomal pathway. This evasion mechanism circumvents NLRP3 inflammasome–driven pyroptotic responses and may favor viral replication.

### RSV inhibits caspase-8–mediated extrinsic apoptosis

Viral infection of immune cells provokes the release of cytokines, including interleukins and TNF, which orchestrate inflammatory and immune defenses against pathogens (*44*). TNF, as an extracellular apoptotic signal, activates extrinsic apoptosis by binding its receptor, assembling the DISC, and engaging caspase-8. This pathway rapidly eliminates infected cells and restricts viral spread (*13*, *45*). Our findings demonstrate that while RSV infection of THP-1 macrophages rapidly induces robust TNF secretion, it fails to promptly activate extrinsic apoptosis (Fig. 1). Instead, the apoptosis induced at the late stage of RSV infection is mediated by the caspase-9–dependent intrinsic pathway (Fig. 3). Previous studies have shown that in *Casp-1*^−/−^ macrophages, inflammasome activation can engage caspase-8 via ASC to promote extrinsic apoptosis (*18*, *46*). Consistently, NLRP3 activation with Nig induced caspase-8–dependent apoptosis in *Casp-1*^−/−^ cells; however, RSV infection did not, despite inflammasome activation and ASC oligomerization (Fig. 3, A to D, and fig. S5). These results suggest that RSV may suppress caspase-8–mediated extrinsic apoptosis.

**Fig. 5.**
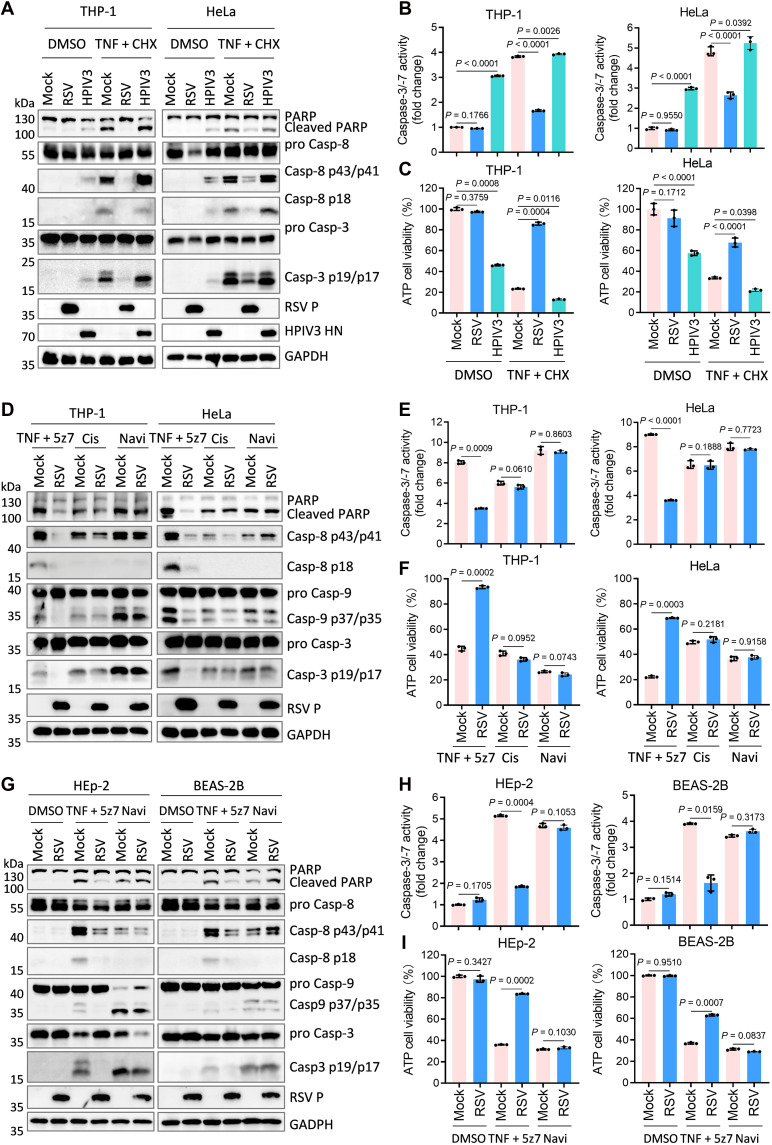
RSV inhibits caspase-8–mediated extrinsic apoptosis in immune cells and nonimmune cells. (**A** to **C**) PMA-differentiated WT THP-1 macrophages or HeLa cells were infected with RSV (MOI = 3.0) or HPIV3 (MOI = 3.0) for 8 hours and then stimulated with TNF (20 ng/ml) plus cycloheximide (CHX; 10 μg/ml) for 4 hours. Cell lysates were analyzed by immunoblots (A), caspase-3/-7 activity assay (B), or cell viability (ATP Glo) evaluation (C). (**D** to **F**) PMA-differentiated WT THP-1 macrophages or HeLa cells were infected with RSV (MOI = 3.0) for 6 to 8 hours and then stimulated with TNF (20 ng/ml) plus 5z-7-oxozeaenol (5z7; 4 μM) for 5 hours, cisplatin (Cis; 60 μg/ml) for 8 hours, or navitoclax (Nav; 50 μM) for 5 hours. Cell lysates were analyzed by immunoblots (D), caspase-3/-7 activity assay (E), or cell viability (ATP Glo) evaluation (F). (**G** to **I**) HEp2 or BEAS-2B were infected with TNF (20 ng/ml) plus 5z7 (4 μM) for 5 hours or Navi (50 μM) for 5 hours. Cell lysates were analyzed by immunoblots (G), caspase-3/-7 activity assay (H), or cell viability (ATP Glo) evaluation (I). Graphs show mean ± SD (*n* = 3 biologically independent experiments). Statistical significance was determined by two-way ANOVA in [(B), (C), (E), (F), (H), and (I)]. Casp, caspase.

To investigate RSV-mediated inhibition of extrinsic apoptosis, THP-1 and HeLa were infected with RSV or human parainfluenza virus type 3 [HPIV3; a positive control virus known to induce apoptosis (*47*, *48*)], followed by stimulation with TNF and cycloheximide (CHX). HPIV3 infection alone triggered apoptosis, whereas RSV did not. Moreover, RSV markedly suppressed apoptosis induced by TNF plus CHX (Fig. 5, A to C). RSV also inhibited extrinsic apoptosis initiated by diverse stimuli, including TNF combined with 5z7, birinapant (Bir), or FasL, but exerted no effect on intrinsic apoptosis elicited by cisplatin (Cis) or Navi (Fig. 5, D to I, and fig. S6, A to F).

Caspase-8 activation can initiate intrinsic apoptosis via BID cleavage (*49*), potentially explaining the observed reduction in caspase-9 cleavage by RSV even upon treatment with extrinsic inducers (Fig. 5, D and G, and fig. S6, A and D). To test this, WT, *Casp-8*^−/−^ or *Casp*-9^−/−^ THP-1 and HeLa cells were generated. To circumvent the inhibitory effect of CHX on viral protein synthesis and minimize potential undesirable effects of Bir, TNF plus 5z7 was used as the extrinsic apoptosis inducer in subsequent experiments. In WT cells, RSV suppressed TNF plus 5z7-induced caspase-9 cleavage; this suppressive effect was lost in *Casp-8*^−/−^ cells (fig. S7, A to E, lanes 1, 2, 5, and 6). Notably, RSV did not inhibit Navi-induced caspase-9 cleavage in any cell line tested (fig. S7, A to E, lanes 3, 4, 7, and 8). These findings indicate that RSV blocks caspase-9 activation by targeting caspase-8–dependent extrinsic pathways. Collectively, our data demonstrate that RSV selectively inhibits the caspase-8–dependent extrinsic apoptotic cascade without affecting caspase-9–mediated intrinsic apoptosis.

### cFLIP mediates inhibition of RSV-induced extrinsic apoptosis

**Fig. 6.**
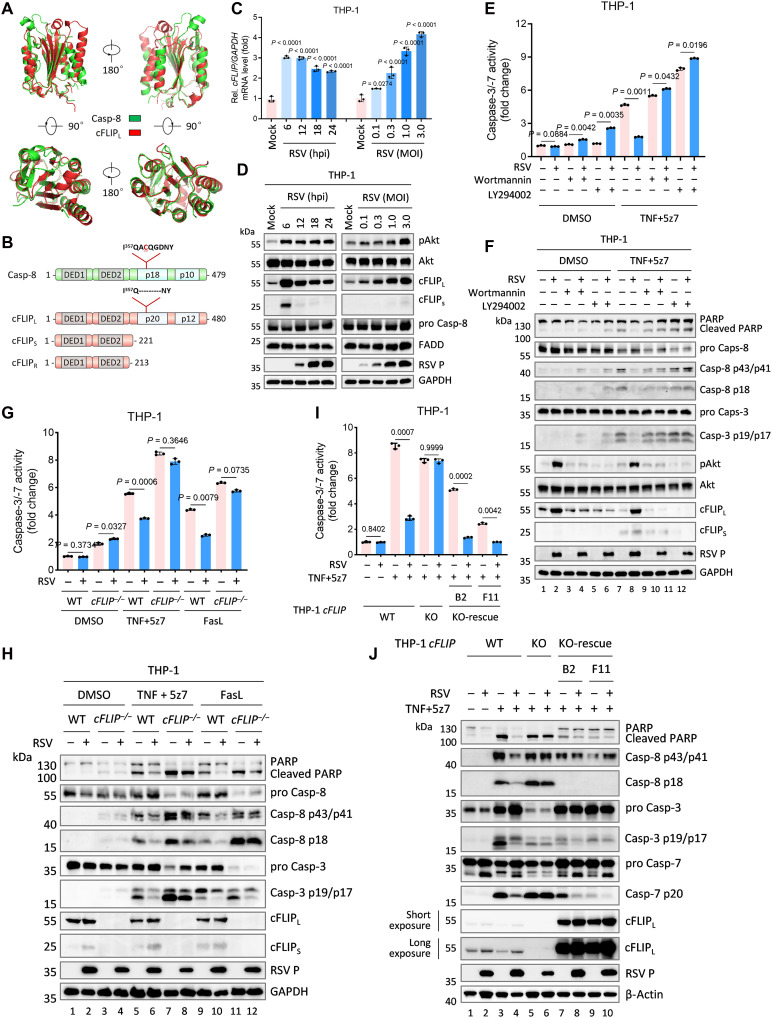
cFLIP is involved in the inhibition of RSV-induced extrinsic apoptosis in macrophages. (**A**) Structural alignment of caspase-8 (PDB ID 6PX9) and cFLIP_L_ (PDB ID 3H13). (**B**) Schematic representation of caspase-8, cFLIP_L_, cFLIP_S_, and cFLIP_R_ domain structures. (**C** and **D**) PMA-differentiated WT THP-1 macrophages were infected with RSV (MOI = 3.0) for indicated times or at different MOI for 12 hours. Cells were collected and RT-qPCR was performed to measure the transcription of indicated genes (C). Cell lysates were analyzed by immunoblots (D). (**E** and **F**) PMA-differentiated WT THP-1 macrophages were infected with RSV (MOI = 3.0) for 7 hours in the presence/absence of wortmannin (200 μM) or LY294002 (10 μM), and then stimulated with TNF (20 ng/ml) plus 5z-7-oxozeaenol (5z7; 4 μM) for 5 hours. Cell lysates were analyzed by caspase-3/-7 activity assay (E) or immunoblots (F). (**G** and **H**) PMA-differentiated WT or *cFLIP*^−/−^ THP-1 macrophages were infected with RSV (MOI = 3.0) for 6 to 7 hours, and then stimulated with TNF (20 ng/ml) plus 5z7 (4 μM) for 5 hours or FasL (200 ng/ml) for 6 hours. Cell lysates were analyzed by caspase-3/-7 activity assay (G) or immunoblots (H). (**I** and **J**) PMA-differentiated WT, *cFLIP* KO, or two *cFLIP* KO-rescue (B2 and F11) THP-1 macrophages were infected with RSV (MOI = 3.0) for 7 hours, and then stimulated with TNF (20 ng/ml) plus 5z7 (4 μM) for 5 hours. Cell lysates were analyzed by caspase-3/-7 activity assay (I) or immunoblots (J). Graphs show mean ± SD (*n* = 3 biologically independent experiments). Statistical significance was determined by two-way ANOVA in [(C), (E), (G), and (I)]. Casp, caspase; hpi, hours postinfection.

During extrinsic apoptosis, engagement of death receptors, including Fas, TNFR1, and TRAILR, by their cognate ligands recruits the adaptor protein FADD (Fas-associated protein with death domain), which assembles with pro–caspase-8, TRADD (TNFR1-associated death domain protein), cFLIP, and other adaptors to form the DISC (*45*). Within the DISC, pro–caspase-8 undergoes autocatalytic activation, initiating the apoptotic cascade. Structurally homologous to pro–caspase-8 but catalytically inactive, cFLIP exists as multiple isoforms: cFLIP_L_, which heterodimerizes with pro–caspase-8 to suppress its activation; cFLIP_S_, which competes for FADD binding to modulate signal transduction; and cFLIP_R_, a T cell–specific isoform, functionally analogous to cFLIP_S_ (*50*) (Fig. 6, A and B).

Previous studies indicate that cFLIP expression and stability are regulated by the nuclear factor κB and PI3K-Akt pathways, both of which can be activated by RSV to modulate cytokine release and apoptosis (*51*, *52*). Consistently, in both THP-1 and HeLa cells, RSV robustly increased Akt phosphorylation and up-regulated *cFLIP* mRNA and protein levels, without affecting FADD or caspase-8 expression (Fig. 6, C and D, and fig. S8, A and B). In addition, we confirmed that RSV up-regulates cFLIP in a broad panel of cell lines (fig. S8C). Inhibition of PI3K with wortmannin or LY294002 abrogated RSV-induced PI3K phosphorylation and cFLIP expression, thereby reversing RSV-mediated inhibition of extrinsic apoptosis (Fig. 6, E and F, and fig. S8, D and E).

To directly test the role of cFLIP, we compared RSV-mediated effects on extrinsic apoptosis in WT and *cFLIP*-deficient (*cFLIP^−/−^*) HeLa cells following stimulation with extrinsic inducers. Apoptosis was evaluated by measuring the cleavage of apoptotic substrates, caspase-3/-7 activity, and nuclear morphology. In WT cells, RSV significantly suppressed extrinsic apoptosis triggered by TNF plus 5z7 or FasL; this suppression was absent in *cFLIP^−/−^* cells (Fig. 6, G and H, and fig. S8, F and G). Reconstitution of *cFLIP* in knockout cells restored RSV-mediated inhibition of apoptosis (Fig. 6, I and J, and fig. S8, H to J). Collectively, these findings demonstrate that RSV up-regulates cFLIP expression via the PI3K-Akt pathway, thereby inhibiting death receptor–mediated extrinsic apoptosis.

### RSV dynamically regulates apoptosis and pyroptosis to facilitate its replication

**Fig. 7.**
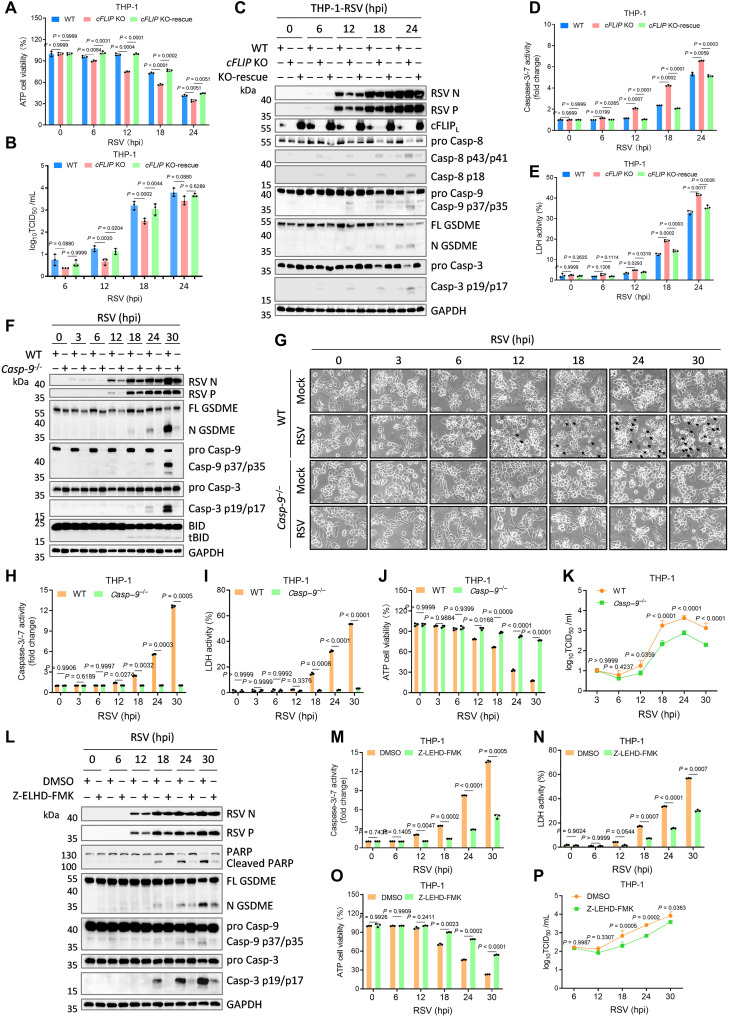
RSV dynamically regulates apoptosis and pyroptosis for self-replication. (**A** to **E**) PMA-differentiated WT, *cFLIP* KO, or *cFLIP* KO-rescue THP-1 macrophages were infected with RSV (MOI = 3.0) for indicated times. The percentage of viable cells was determined through cell viability (ATP Glo) evaluation (A). Cell lysates were analyzed by immunoblots (C) and caspase-3/-7 activity assay (D), and supernatants were harvested for measurements of the viral titers with standard TCID_50_ assays (B) and LDH release (E). (**F** to **K**) PMA-differentiated WT or *Casp-9* KO THP-1 macrophages were infected with RSV (MOI = 3.0) for indicated times. Cell lysates were analyzed by immunoblots (F) and caspase-3/-7 activity assay (H). Arrows indicate pyroptotic or apoptotic cells under microscopy (G). The percentage of viable cells was determined through cell viability (ATP Glo) evaluation (J). Cell supernatants were harvested for measurements of LDH release (I) and the viral titers with standard TCID_50_ assays (K). (**L** to **P**) PMA-differentiated WT THP-1 macrophages were infected with RSV (MOI = 3.0) for indicated times in the presence/absence of the caspase-9 inhibitor Z-ELHD-FMK (30 μM). Cell lysates were analyzed by immunoblots (L) and caspase-3/-7 activity assay (M). Cell supernatants were harvested for measurements of the viral titers with standard LDH release (N) and TCID_50_ assays (P). The percentage of viable cells was determined through cell viability (ATP Glo) evaluation (O). Graphs show mean ± SD (*n* = 3 biologically independent experiments). Statistical significance was determined by two-way ANOVA in [(A), (B), (D), (E), (H) to (K), and (M) to (P)]. Casp, caspase; FL-GSDME, full-length GSDME; N-GSDME, N-terminal GSDME; hpi, hours postinfection.

The regulation of cell death by RSV is critical for viral replication and propagation. As shown in Fig. 6, disruption of RSV-mediated inhibition of extrinsic apoptosis, either by PI3K inhibition or *cFLIP* deficiency, markedly reduced viral protein expression, whereas restoration of *cFLIP* rescued viral production. These findings indicate that RSV exploits cFLIP to suppress extrinsic apoptosis, thereby delaying host cell death and favoring viral replication. To further dissect this relationship, we monitored cell death, virion release, and viral protein expression at various time points after RSV infection in WT, *cFLIP*-deficient, and *cFLIP*-rescued THP-1 and HeLa cells. In *cFLIP*-deficient THP-1 cells, RSV infection rapidly induced cell death, resulting in reduced viral replication, as demonstrated by ATP viability assays and viral titration (Fig. 7, A and B). Western blotting and caspase-3/-7 activity assays revealed that *cFLIP* ablation led to early activation of caspase-8 and caspase-3 within 6 hours postinfection, triggering apoptosis and diminishing viral protein expression (Fig. 7, C and D). Moreover, at the later stages of infection, RSV induced caspase-9–mediated intrinsic apoptosis and GSDME-dependent secondary pyroptosis, thereby exacerbating cell death (Fig. 7, A, C, and E). In contrast, in WT or *cFLIP*-rescued THP-1 cells, RSV ultimately induced intrinsic apoptosis and GSDME-mediated secondary pyroptosis at 18 hours postinfection, resulting in comparable cell death and viral titers (Fig. 7, A to E).

In HeLa cells, which lack the NLRP3 inflammasome, RSV cannot trigger intrinsic apoptosis or GSDME-mediated pyroptosis via caspase-1. However, *cFLIP* knockout enabled RSV to induce extrinsic apoptosis, leading to a sustained reduction in viral replication (fig. S9, A and B), likely due to viral up-regulation of Fas and the release of Fas-mediated apoptotic activity upon loss of *cFLIP* (*53*). In WT and *cFLIP*-rescued HeLa cells, RSV did not efficiently induce apoptosis (fig. S9, A, C, and D), whereas *cFLIP*-deficient cells displayed activation of caspase-8 and subsequent cleavage of caspase-9 and GSDME, culminating in cell death (fig. S9, A, C to E). Morphological analysis further confirmed that RSV infection triggered widespread cell death in *cFLIP* knockout HeLa cells, in contrast to the syncytium formation observed in WT and *cFLIP*-rescued cells, which facilitates viral spread (*54*) (fig. S9F).

In contrast to cFLIP-dependent suppression of extrinsic apoptosis, loss of *caspase-9*, *BID*, or *APAF1* suppressed intrinsic apoptosis at later stages of RSV infection, paradoxically reducing viral protein expression (Fig. 3). We hypothesize that blocking intrinsic apoptosis and GSDME-mediated secondary pyroptosis hinders the rapid release of newly formed virions, thus restricting viral dissemination. To test this, we infected WT and *Casp-9^−/−^* THP-1 cells with RSV. Western blot and viability analyses showed that *caspase-9* deficiency nearly abrogated RSV-induced cleavage of caspase-3 and GSDME, markedly reducing cell death and subsequent viral protein expression and virion release (Fig. 7, F to K). Consistently, pharmacological inhibition of caspase-9 with Z-LEHD-FMK reproduced these findings, confirming that caspase-9–mediated cell death is exploited by RSV to promote viral release (Fig. 7, L to P).

Collectively, our data reveal a crucial relationship between RSV replication and PCD. RSV up-regulates cFLIP to suppress extrinsic apoptosis, supporting viral replication, while later activating the Casp-1–BID–APAF1–Casp-9 axis to trigger GSDME-mediated secondary pyroptosis and facilitate virion release. This regulatory network not only promotes viral propagation but also drives vigorous host inflammatory responses. Thus, targeting cFLIP or caspase-9 offers a potential strategy to limit RSV replication and dissemination, while mitigating virus-induced immunopathology.

## DISCUSSION

In this study, we found that RSV infection induces inflammatory cytopathy and up-regulates *IL-1*β mRNA levels in various cell types, and also triggers substantial release of TNF and IL-6 from THP-1 macrophages. In contrast, THP-1 macrophages only released minimal mature IL-1β, and exhibited negligible cell death during early RSV infection. (Fig. 1, A to F, and fig. S1). These observations suggest RSV actively suppresses initial inflammasome responses and pyroptotic cell death. Although macrophages typically mount rapid inflammasome-dependent pyroptosis and cytokine release upon viral infection, RSV-infected cells showed delayed cell death and inflammatory cytokine secretion. While prior work demonstrates RSV can up-regulate NLRP3 inflammasome components (*55*, *56*), our pharmacological and genetic analyses confirm rapid NLRP3 activation and IL-1β processing by RSV (Fig. 1, G to I, and fig. S2, E to H). However, despite early GSDMD cleavage, neither pyroptosis nor substantial IL-1β release occurred (Fig. 1, D to F and I). Only at later stages (>18 hours postinfection) did RSV induce apoptosis, leading to caspase-3–mediated noncanonical cleavage of GSDMD and subsequent cell lysis (Fig. 1, I to L). These findings suggest RSV initially restrains N-GSDMD–driven pyroptosis but later promotes IL-1β release via apoptosis-mediated cell death, potentially explaining the mild early and severe late clinical course of RSV infection (*28*, *29*).

Pyroptosis can be executed not only by caspase-1–cleaved N-GSDMD but also via caspase-3–mediated cleavage of GSDME during apoptosis (*36*). We found that RSV-induced plasma membrane rupture and IL-1β release were unaffected by *GSDMD* knockout, but abolished upon *GSDME* deletion (Fig. 2, L to O), indicating that RSV promotes GSDME-dependent pyroptosis for IL-1β secretion in late infection. GSDME cleavage was contingent on upstream activation of caspase-3, which can be triggered by both extrinsic and intrinsic pathways (*12*). Using *Casp-8*^−/−^ or *Casp-9*^−/−^ THP-1 macrophages, we established that RSV-induced GSDME cleavage proceeds specifically via the intrinsic pathway (Fig. 3, A to D, fig. S3). Notably, *Casp-1*^−/−^ cells failed to undergo apoptosis or pyroptosis in response to RSV infection (Fig. 3, A to D), implicating caspase-1 in both intrinsic apoptosis and GSDME-dependent pyroptosis. Previous work shows that in *GSDMD*-deficient contexts, caspase-1 can initiate mitochondrial damage and apoptosis by cleaving BID (*17*, *37*). Using genetic approaches, our data delineate a unique pyroptotic cascade in RSV-infected macrophages: NLRP3 inflammasome activation leads to GSDME cleavage via the Casp-1–BID–APAF1–Casp-9 axis (Fig. 3, I to L).

While canonical pyroptosis is activated through caspase-1–dependent GSDMD cleavage (*14*), whether RSV actively restrains N-GSDMD–mediated pyroptosis to favor GSDME-dependent execution remained unresolved. In RSV-infected *Casp-9*^−/−^ macrophages treated with inflammasome inducers, RSV enhanced GSDMD and IL-1β cleavage but suppressed N-GSDMD–driven pyroptosis and IL-1β release (Fig. 4, A to C). Recent studies show that N-GSDMD palmitoylation by ZDHHC5/9 is essential for pyroptosis (*40*, *41*). Our analyses identified RSV-induced down-regulation of ZDHHC9 (Fig. 4, D to E). Inhibition of degradation pathways revealed that RSV promotes ZDHHC9 degradation through the autophagy-lysosomal pathway, thus suppressing N-GSDMD–mediated pyroptosis and mature IL-1β release (Fig. 4, F to H). While RSV is known to leverage selective autophagy to degrade host factors and enhance replication (*43*), precise regulatory details of pyroptosis suppression remain to be clarified. Notably, restoration of ZDHHC9 and N-GSDMD function markedly inhibited viral protein expression (Fig. 4H), indicating that RSV might specifically suppress N-GSDMD–mediated pyroptosis to facilitate replication.

Upon pathogen recognition, innate immune cells secrete inflammatory cytokines in a coordinated response. RSV induces TNF production via TLR signaling (*35*, *57*), TNF then stimulates extrinsic apoptosis to constrain viral expansion (*45*). Despite known RSV suppression of extrinsic apoptosis, underlying mechanisms have been unclear (*52*). Here, we demonstrate that RSV up-regulates cFLIP via PI3K-Akt activation (Fig. 6, C to F, and fig. S8, A to E); cFLIP, a structural homolog of caspase-8, modulates caspase-8 through heterodimerization (*50*). Through knockout and rescue experiments, we identify cFLIP as the key mediator of RSV-dependent inhibition of extrinsic apoptosis (Fig. 6, G to J, and fig. S8, F to J).

It has been reported that when caspase-8 activity is inhibited, TNF stimulation recruits FADD, leading to the phosphorylation of RIPK1, RIPK3, and MLKL and the triggering of necroptosis (*9*). However, our results show that although *caspase-8* knockout switches the cell death induced by TNF plus SM-164 (an IAP antagonist) from apoptosis to necroptosis, RSV, similar to the effect of TNF plus 5z7, fails to induce necroptosis in *Casp-8*^−/−^ cells (fig. S3, E to H). We speculate that the high levels of cIAP2/XIAP in THP-1 cells may sequester RIPK1 within complex I, thereby impeding its release and subsequent polymerization with RIPK3 following RSV infection or TNF plus 5z-7 stimulation. Studies have reported that, in addition to inducing caspase-8–mediated extrinsic apoptosis, TNF plus 5z7 can also trigger TRIF-dependent death in macrophages (*58*). Conversely, it has been reported that RSV, in addition to suppressing extrinsic apoptosis induced by the aforementioned agents, also down-regulates TLR4 expression within the TRIF-dependent cell death pathway (*59*), which may lead to the indirect inhibition of multiple death signaling routes. These intricate signaling mechanisms likely explain why neither RSV infection nor TNF plus 5z7 treatment induces necroptosis in caspase-8–deficient cells.

Virus-induced cell death shapes pathogenesis bidirectionally: Premature cell death restricts replication, whereas delayed death enhances virion release (*13*, *60*). Understanding these dynamics is critical, as RSV primarily targets airway epithelium, influencing disease severity. Our results show that reinstating extrinsic apoptosis with *cFLIP* knockout or restoring N-GSDMD–mediated pyroptosis (via inhibition of autophagy-lysosomal degradation) suppresses RSV protein levels (Figs. 4H and 7, A to D, and fig. S9). In contrast, intrinsic apoptosis and GSDME-mediated secondary pyroptosis boost viral protein expression and titers, as seen in WT but not *Casp9*^−/−^ macrophages (Figs. 3 and 7 and fig. S3). Thus, RSV appears to selectively modulate distinct cell death programs to optimize replication. Genetic manipulation and complementation experiments allowed us to elucidate this dynamic mechanism: RSV adopts a biphasic strategy (fig. S10): early suppression of host defenses through cFLIP up-regulation and ZDHHC9 degradation to inhibit extrinsic apoptosis and N-GSDMD–mediated pyroptosis, followed by late-stage activation of the Casp-1–BID–APAF1–Casp-9 axis to induce GSDME-mediated secondary pyroptosis, promoting virion release.

Collectively, our findings reveal a direct mechanistic link between RSV replication and host cell death regulation. Rapid induction of infected cell death can limit viral replication, whereas suppression of caspase-1–driven mitochondrial apoptosis may serve as an alternative means to constrain viral dissemination, necessitating precise control over NLRP3 activation.

## MATERIALS AND METHODS

### Plasmids, antibodies and reagents

The plasmids, oligonucleotides, antibodies and reagents used in this study are all listed in tables S1 to S3.

### Cell cultures and treatment

THP-1 cells (American Type Culture Collection, catalog no. TIB-202) were cultivated in RPMI 1640 (Gibco) supplemented with 10% fetal bovine serum (FBS; Wisent) and 1% penicillin-streptomycin (Gibco). HEK293T, HeLa, Vero, HEp-2, BEAS-2B, and A549 cells were obtained from China Center for Type Culture Collection and maintained in Dulbecco’s modified Eagle’s medium (Gibco) supplemented with 10% FBS (Wisent) and 1% penicillin-streptomycin (Gibco). Cells were cultured at 37°C in a 5% CO_2_ incubator. THP-1 cells were differentiated into macrophages by treatment with PMA (100 ng/ml) for 24 hours at 37°C. Then, the cells were cultured in fresh medium for another 12 hours.

Transient transfection was performed using NEOFECT DNA transfection reagent according to the manufacturer’s instructions. For NLRP3 inflammasome activation, THP-1 cells were primed with ultrapure LPS (1 μg/ml) for 4 hours and then stimulated with 2.5 μM Nig for 2 hours. For AIM2 inflammasome activation, LPS-primed THP-1 cells were transfected with poly(dA:dT) (2 μg/ml) for 6 hours using Lipofectamine LTX with Plus Reagent. For NLRP1 inflammasome activation, THP-1 cells were stimulated with 20 μM Val-boroPro mesylate (Val-boro) for 6 hours. For PCD regulation, THP-1 cells were treated with 20 μM MCC950 or 50 μM VX765 for 1 hour before infection to inhibit NLRP3 or caspase-1, respectively. Z-VAD-FMK (30 μM), 30 μM necrostatin-1, or 10 μM Fer-1 were added before infection for 1 hour to inhibit caspase-dependent cell death, necroptosis, or ferroptosis, respectively. To induce extrinsic apoptosis, cells were treated with FasL (200 ng/ml) for 6 hours, TNF-α (20 ng/ml) plus CHX (10 μg/ml), 4 μM 5z7, or Bir (20 ng/ml) for 4 hours. To induce intrinsic apoptosis, cells were treated with 50 μM Navi for 5 hours or Cis (60 μg/ml) for 8 hours. To induce necroptosis, cells were treated with TNF-α (100 ng/ml) plus 1 μM SM-164 for 12 hours. To inhibit caspase-9 activation, THP-1 cells were treated with 30 μM Z-LEHD-FMK for 1 hour before infection.

THP-1 cells and HeLa cells were treated with 200 μM wortmannin or 10 μM LY294002 for 1 hour before infection to inhibit PI3K-Akt signaling pathway. THP-1 cells were treated with 1 mM 3-MA, 1 μM CQ, 1 μM MG132, or 100 nM Bort for 1 hour before infection to inhibit protein degradation.

### Viral infection and titration

Human RSV (A2 strain) and HPIV3 used in the experiments were stored by our laboratory. To generate large stocks of RSV, HEp2 cells were infected with RSV at MOI of 0.01 for 2 hours, then incubated sequentially in a fresh medium containing 5% FBS for another 5 days. The supernatant was harvested and centrifuged at 6000 rpm at 4°C for 10 min to remove cellular debris. To obtain a high-titer viral suspension, the virus supernatant was concentrated with polyethylene glycol (PEG) 6000 (*61*). In brief, the virus supernatant was precipitated by stirring with 10% PEG6000 overnight at 4°C, then pelleted by centrifugation at 4300*g* at 4°C for 30 min. The pellets were resuspended in NT buffer (0.15 M NaCl and 0.05 M tris, pH 7.5) and aliquoted into tubes and stored in liquid nitrogen.

Virus titers were determined by the median tissue culture infectious dose (TCID_50_) assay using Vero cells. To be specific, Vero cells were initially seeded into 96-well plates at a controlled density, typically falling within the range of 20 to 30%. Following this setup, a serial dilution of the virus sample was added to the 96-well plates. Two hours later, the virus sample was replaced by a fresh medium containing 5% FBS. Then, cells were incubated for another 5 days. The assessment involved the careful observation of CPEs in each well at various dilutions. The count of wells exhibiting these CPE served as the basis for quantifying the viral titers. The calculations were performed in adherence to the Karber method.

### Generation of stably expressing and knockout (KO) cell lines

To generate the knockout cell lines, sgRNA sequences for target proteins were cloned into lentiCRISPRv2. HEK293T cells were cotransfected with lentiCRISPRv2, pSPAX2, and pMD2.G at a ratio of 5:3:2 using the NEOFECT DNA transfection reagent following the manufacturer’s instructions. Forty-eight hours after the transfection, cell supernatant containing the lentivirus was filtered with a 0.45-μm pore filter (Millipore) and collected to infect THP-1 or HeLa cells in the presence of polybrene (8 μg/ml) for another 24 hours. Mixed populations of infected cells were selected with puromycin (2 μg/ml) for 1 week and then harvested to detect the deletion of target proteins. The single clones were screened in 96-well plates for another 15 to 20 days or longer and validated by immunoblot analysis using an available antibody.

To generate reconstituted cell lines, the pCDH-CMV-IRES-MCS-SF-BLAST lentiviral vector containing the desired gene, together with the packaging plasmids pMD2.G and pSPAX2, were cotransfected into HEK293T cells at a ratio of 5:3:2. Forty-eight hours later, cell supernatant was harvested and used to infect THP-1 or HeLa cells. After incubation for 24 hours in the presence of polybrene (8 μg/ml), the cells were incubated with fresh medium and selected with blasticidin S (10 μg/ml). Single-cell clones were obtained by limiting dilution. Protein expression of selected single-cell clones were tested by immunoblot analysis.

### Immunoblotting analysis

Cells were lysed with lysis buffer [150 mM NaCl, 50 mM Tris-HCl (pH 7.4), 1% Triton X-100, 1 mM EDTA (pH 8.0), 0.1% SDS, 0.1% protease inhibitor cocktail, and 0.5 mM phenylmethylsulfonyl fluoride] for 30 min on ice. Protein concentration was determined by modified Bradford reagent. Equivalent amounts of proteins were boiled with 5× SDS loading buffer [250 mM Tris-HCl (pH 6.8), 10% SDS, 50% glycerol, 0.5% bromophenol blue, and 5 M dithiothreitol] at 100°C for 10 min and then electrophoresed in SDS–polyacrylamide gel electrophoresis and transferred to a nitrocellulose membrane (Millipore). Nonspecific antibody binding sites were blocked with 5% skim milk in phosphate-buffered saline (PBS) with 0.1% Tween 20, then incubated with the indicated primary antibodies. Protein bands were detected by Clarity Western ECL Substrate (Bio-Rad) using ChemiDoc MP imaging system (Bio-Rad) and Image Lab version 6.1.0 software.

Supernatant of the cultured cells was harvested and centrifuged at 13,000 rpm at 4°C for 1 min to remove cellular debris. Then, the supernatant was centrifuged at 14,000*g* for 15 min each time by Amicon Ultra (Millipore, UFC500324) for protein concentrate. The concentrated supernatant was mixed with SDS loading buffer for Western blotting analysis.

### Immunofluorescence imaging

The PMA-differentiated THP-1 cells were seeded at 500,000 cells per well of a six-well plate. After treatment, cells were fixed with 4% paraformaldehyde for 15 min at room temperature (RT). Then, 5 μM Sytox Green and 1× Hoechst 33342 were used to stain cracked cell and nuclear DNA, respectively. Fluorescence images were acquired by using Leica DMi8 Inverted Fluorescence Microscope. ImageJ 1.54m was used for automated counting of Sytox Green– and Hoechst-positive nuclei.

To visualize apoptosis, HeLa cells were seeded at 5000 cells per well of a six-well plate and infected or treated on the following day. Cells were fixed with 4% paraformaldehyde for 15 min at RT and then stained with 1× Hoechst 33342 for 10 min. Fluorescence images were taken with Leica DMi8 Inverted Fluorescence Microscope and analyzed by Leica Application Suite Advanced Fluorescence Lite.

### Caspase-3/-7 activity assay

Caspase-3/-7 activities were determined by using the caspase-Glo 3/7 assay system (G8092, Promega) according to the manufacturer’s instructions. Cells were plated in each well of six-well plates and were incubated overnight. After treatment, cells were collected and lysed. An equal volume of caspase-Glo 3/7 reagent was added to the lysate and shaken at 500 rpm for 30 min at RT. Luminescent recording was performed with SpectraMAX iD3 Multi-mode microplate reader (Sunnyvale, CA, USA).

### LDH and cell viability assay

Cells were seeded in each well of 96-well plates and were incubated overnight. After treatment, the LDH release and ATP cell viability were detected by the CytoTox 96 Non-Radioactive Cytotoxicity Assay kit (G1780, Promega) and the Cell Titer-Glo Luminescent Cell Viability Assay kit (G7571, Promega), respectively, following the manufacturer’s instructions.

### Enzyme-linked immunosorbent assay for cytokine quantification

Cell-free supernatant was collected for quantitative detection of cytokines including IL-1β, IL-18, TNF-α, or IL-6 with commercial enzyme-linked immunosorbent assay (ELISA) kits according to the manufacturers’ instructions. Human IL-1β (catalog no. 437015), IL-6 (catalog no. 430504), and TNF-α (catalog no. 430204) ELISA kits were purchased from BioLegend, and human IL-18 ELISA kit (catalog no. DY318-05) was purchased from R&D Systems.

### ASC oligomerization and speck formation assay

The PMA-differentiated THP-1 cells were lysed in lysis buffer [20 mM Tris-HCl (pH 7.4), 150 mM NaCl, 1 mM EDTA, 1% Triton X-100, 0.1% (wt/vol) SDS, and protease inhibitor cocktail] for 30 min on ice with gentle rocking every 10 min. Lysates were centrifuged at 6500 rpm for 15 min at 4°C. The supernatant of the lysates was mixed with SDS loading buffer for Western blotting analysis. The pellets of the lysates were washed with PBS for three times and cross-linked using fresh dextran sulfate sodium (2 mM, Sigma-Aldrich) at 37°C for 30 min. The cross-linked pellets were then centrifuged at 6000 rpm for 10 min. The precipitate mixed with 1× SDS loading buffer for Western blotting analysis.

To detect the formation of ASC speck, the PMA-differentiated THP-1 cells on coverslips in 24-well plates were infected for specific time. Thereafter, cells were washed three times in 1 × PBS and fixed in 4% paraformaldehyde for 15 min at RT. Then, cells were washed in 1 × PBS and permeabilized with 0.5% Triton X-100 for 5 min. After three washes, the cells were blocked with 2% bovine serum albumin (BSA) for 30 min, subsequently incubated in indicated primary antibodies (anti-ASC: 1:200; anti-RSV N: 1:500) diluted in 1% BSA for 4 hours at 4°C. Cells were then washed in 1× PBS again and incubated with Alexa Fluor 488– or Alexa Fluor 647–labeled secondary antibody (1:500) for 1 hour in the dark. Cells were then washed in 1× PBS and counterstained with 4′,6-diamidino-2-phenylindole for 10 min. After successive washing with 1× PBS, the coverslips were mounted onto glass slides using Prolong Diamond Antifade Mountant. Confocal images were taken with Leica confocal microscope and analyzed by Leica Application Suite Advanced Fluorescence Lite.

### Quantitative PCR analysis

Total RNA was extracted with TRIzol reagent and reverse transcribed using a 1st Strand cDNA Synthesis Kit (Yeasen). Real-time qPCR was performed using Hieff qPCR SYBR Green Master Mix (Yeasen) in a CFX96 Touch real-time PCR detection system (Bio-Rad). The quantitative expression of targeted gene was calculated relative to glyceraldehyde-3-phosphate dehydrogenase and normalized to control samples. The primers were designed by National Center for Biotechnology Information or based on references. The sequences were as follows: *IL-1*β, (forward) 5′-CACGATGCACCTGTACGATCA-3′ and (reverse) 5′-GTTGCTCCATATCCTGTCCCT-3′; RSV *N*, (forward) 5′-CTATGGTGCAGGGCAAGTGA-3′ and (reverse) 5′-GAATCCTGCTTCACCACCCA-3′; *cFLIP*, (forward) 5′-ATGCTGCTCTTTTTGTGCCG-3′ and (reverse) 5′-GGTGGGTCTCCACAGCTTTT-3′; *ZDHHC5*, (forward) 5′-CACCTGCCGCTTTTACCGT-3′ and (reverse) 5′-CGGCGACCAATACAGTTATTCAC-3′; *ZDHHC9*, (forward) 5′-TCGGGCGCTACCAGATGAA-3′ and (reverse) 5′-GGGCAGTGATGGTCGAAGC-3′; *ZDHHC17*, (forward) 5′-GATGTACGGCAACCGGACAAA-3′ and (reverse) 5′-TGATCCCAATAGCACCTTTCG-3′; *ZDHHC20*, (forward) 5′-CGCACCCACGTTTTCATACG-3′ and (reverse) 5′-TCTGGCATACTCATTCTGGTTTG-3′; and *GAPDH*, (forward) 5′-AAGGCTGTGGGCAAGG-3′ and (reverse) 5′-TGGAGGAGTGGGTGTCG-3′.

### Growth curves

Cells were seeded in 12-well plates and infected by RSV (MOI = 3). Two hours postinfection, cells were washed twice with PBS, and a fresh medium was added. Supernatant samples were harvested at different times post infection, and viral titers were determined on Vero cells using the TCID_50_ assay. Cell samples were collected and lysed for Western blotting detection.

### Structural alignment

The structures were obtained from Protein Data Bank (PDB) (PDB ID 6PX9 for caspase-8 and 3H13 for cFLIP_L_). All figures for presenting the structures were prepared and analyzed using PyMOL version 1.8.x.

### Statistical analysis

All statistical analyses were performed using GraphPad Prism 9.5. All experiments were repeated at least three times and data are shown as mean ± SD of indicated number of independent wells. Statistical significance was calculated using either unpaired two-tailed Student’s *t* test for comparison between two groups, one-way analysis of variance (ANOVA) with post hoc Dunnett’s test for comparisons among multiple groups with a single control, or two-way ANOVA with post hoc Bonferroni’s test for comparisons among different groups. In all tests, *P* > 0.05 was considered statistically nonsignificant and *P* < 0.05 was considered statistically significant. No statistical method was used to predetermine sample size, but our sample sizes are similar to those reported in previous publications. For all experiments, samples were randomly allocated to groups.

## References

[1] Y. Duan, Z. Liu, N. Zang, B. Cong, Y. Shi, L. Xu, M. Jiang, P. Wang, J. Zou, H. Zhang, Z. Feng, L. Feng, L. Ren, E. Liu, Y. Li, Y. Zhang, Z. Xie, Landscape of respiratory syncytial virus. Chin. Med. J. (Engl.) 137, 2953–2978 (2024). 39501814 10.1097/CM9.0000000000003354 PMC11706595

[2] M. Hu, M. A. Bogoyevitch, D. A. Jans, Impact of respiratory syncytial virus infection on host functions: Implications for antiviral strategies. Physiol. Rev. 100, 1527–1594 (2020). 32216549 10.1152/physrev.00030.2019

[3] J. G. Wildenbeest, D. M. Lowe, J. F. Standing, C. C. Butler, Respiratory syncytial virus infections in adults: A narrative review. Lancet Respir. Med. 12, 822–836 (2024). 39265602 10.1016/S2213-2600(24)00255-8

[4] A. R. Falsey, P. A. Hennessey, M. A. Formica, C. Cox, E. E. Walsh, Respiratory syncytial virus infection in elderly and high-risk adults. N. Engl. J. Med. 352, 1749–1759 (2005). 15858184 10.1056/NEJMoa043951

[5] N. I. Mazur, M. T. Caballero, M. C. Nunes, Severe respiratory syncytial virus infection in children: Burden, management, and emerging therapies. Lancet (London, England) 404, 1143–1156 (2024). 39265587 10.1016/S0140-6736(24)01716-1

[6] Y. Li, X. Wang, D. M. Blau, M. T. Caballero, D. R. Feikin, C. J. Gill, S. A. Madhi, S. B. Omer, E. A. F. Simões, H. Campbell, A. B. Pariente, D. Bardach, Q. Bassat, J. S. Casalegno, G. Chakhunashvili, N. Crawford, D. Danilenko, L. A. H. Do, M. Echavarria, A. Gentile, A. Gordon, T. Heikkinen, Q. S. Huang, S. Jullien, A. Krishnan, E. L. Lopez, J. Markić, A. Mira-Iglesias, H. C. Moore, J. Moyes, L. Mwananyanda, D. J. Nokes, F. Noordeen, E. Obodai, N. Palani, C. Romero, V. Salimi, A. Satav, E. Seo, Z. Shchomak, R. Singleton, K. Stolyarov, S. K. Stoszek, A. von Gottberg, D. Wurzel, L. M. Yoshida, C. F. Yung, H. J. Zar, H. Nair, Global, regional, and national disease burden estimates of acute lower respiratory infections due to respiratory syncytial virus in children younger than 5 years in 2019: A systematic analysis. Lancet (London, England) 399, 2047–2064 (2022). 35598608 10.1016/S0140-6736(22)00478-0 PMC7613574

[7] T. J. Ruckwardt, K. M. Morabito, B. S. Graham, Immunological lessons from respiratory syncytial virus vaccine development. Immunity 51, 429–442 (2019). 31533056 10.1016/j.immuni.2019.08.007

[8] Y. Ouyang, H. Liao, Y. Hu, K. Luo, S. Hu, H. Zhu, Innate immune evasion by human respiratory syncytial virus. Front. Microbiol. 13, 865592 (2022). 35308390 10.3389/fmicb.2022.865592 PMC8931408

[9] Y. Ai, Y. Meng, B. Yan, Q. Zhou, X. Wang, The biochemical pathways of apoptotic, necroptotic, pyroptotic, and ferroptotic cell death. Mol. Cell 84, 170–179 (2024). 38181758 10.1016/j.molcel.2023.11.040

[10] E. Lee, C. H. Song, S. J. Bae, K. T. Ha, R. Karki, Regulated cell death pathways and their roles in homeostasis, infection, inflammation, and tumorigenesis. Exp. Mol. Med. 55, 1632–1643 (2023). 37612410 10.1038/s12276-023-01069-y PMC10474065

[11] K. Newton, A. Strasser, N. Kayagaki, V. M. Dixit, Cell death. Cell 187, 235–256 (2024). 38242081 10.1016/j.cell.2023.11.044

[12] S. Kesavardhana, R. K. S. Malireddi, T. D. Kanneganti, Caspases in cell death, inflammation, and pyroptosis. Annu. Rev. Immunol. 38, 567–595 (2020). 32017655 10.1146/annurev-immunol-073119-095439 PMC7190443

[13] N. Kayagaki, J. D. Webster, K. Newton, Control of cell death in health and disease. Annu. Rev. Pathol. 19, 157–180 (2024). 37788577 10.1146/annurev-pathmechdis-051022-014433

[14] X. Liu, S. Xia, Z. Zhang, H. Wu, J. Lieberman, Channelling inflammation: Gasdermins in physiology and disease. Nat. Rev. Drug Discov. 20, 384–405 (2021). 33692549 10.1038/s41573-021-00154-z PMC7944254

[15] S. M. Man, R. Karki, T. D. Kanneganti, Molecular mechanisms and functions of pyroptosis, inflammatory caspases and inflammasomes in infectious diseases. Immunol. Rev. 277, 61–75 (2017). 28462526 10.1111/imr.12534 PMC5416822

[16] V. A. Rathinam, K. A. Fitzgerald, Inflammasome complexes: Emerging mechanisms and effector functions. Cell 165, 792–800 (2016). 27153493 10.1016/j.cell.2016.03.046 PMC5503689

[17] K. Tsuchiya, S. Nakajima, S. Hosojima, D. Thi Nguyen, T. Hattori, T. Manh Le, O. Hori, M. R. Mahib, Y. Yamaguchi, M. Miura, T. Kinoshita, H. Kushiyama, M. Sakurai, T. Shiroishi, T. Suda, Caspase-1 initiates apoptosis in the absence of gasdermin D. Nat. Commun. 10, 2091 (2019). 31064994 10.1038/s41467-019-09753-2 PMC6505044

[18] R. Pierini, C. Juruj, M. Perret, C. L. Jones, P. Mangeot, D. S. Weiss, T. Henry, AIM2/ASC triggers caspase-8-dependent apoptosis in Francisella-infected caspase-1-deficient macrophages. Cell Death Differ. 19, 1709–1721 (2012). 22555457 10.1038/cdd.2012.51 PMC3438500

[19] V. Sagulenko, S. J. Thygesen, D. P. Sester, A. Idris, J. A. Cridland, P. R. Vajjhala, T. L. Roberts, K. Schroder, J. E. Vince, J. M. Hill, J. Silke, K. J. Stacey, AIM2 and NLRP3 inflammasomes activate both apoptotic and pyroptotic death pathways via ASC. Cell Death Differ. 20, 1149–1160 (2013). 23645208 10.1038/cdd.2013.37 PMC3741496

[20] P. Orning, D. Weng, K. Starheim, D. Ratner, Z. Best, B. Lee, A. Brooks, S. Xia, H. Wu, M. A. Kelliher, S. B. Berger, P. J. Gough, J. Bertin, M. M. Proulx, J. D. Goguen, N. Kayagaki, K. A. Fitzgerald, E. Lien, Pathogen blockade of TAK1 triggers caspase-8-dependent cleavage of gasdermin D and cell death. Science (New York, N.Y.) 362, 1064–1069 (2018). 30361383 10.1126/science.aau2818 PMC6522129

[21] J. Sarhan, B. C. Liu, H. I. Muendlein, P. Li, R. Nilson, A. Y. Tang, A. Rongvaux, S. C. Bunnell, F. Shao, D. R. Green, A. Poltorak, Caspase-8 induces cleavage of gasdermin D to elicit pyroptosis during Yersinia infection. Proc. Natl. Acad. Sci. U.S.A. 115, E10888–E10897 (2018). 30381458 10.1073/pnas.1809548115 PMC6243247

[22] Y. Wang, W. Gao, X. Shi, J. Ding, W. Liu, H. He, K. Wang, F. Shao, Chemotherapy drugs induce pyroptosis through caspase-3 cleavage of a gasdermin. Nature 547, 99–103 (2017). 28459430 10.1038/nature22393

[23] C. Rogers, T. Fernandes-Alnemri, L. Mayes, D. Alnemri, G. Cingolani, E. S. Alnemri, Cleavage of DFNA5 by caspase-3 during apoptosis mediates progression to secondary necrotic/pyroptotic cell death. Nat. Commun. 8, 14128 (2017). 28045099 10.1038/ncomms14128 PMC5216131

[24] C. Y. Taabazuing, M. C. Okondo, D. A. Bachovchin, Pyroptosis and apoptosis pathways engage in bidirectional crosstalk in monocytes and macrophages. Cell Chem. Biol. 24, 507–514.e4 (2017). 28392147 10.1016/j.chembiol.2017.03.009 PMC5467448

[25] C. D. Russell, S. A. Unger, M. Walton, J. Schwarze, The human immune response to respiratory syncytial virus infection. Clin. Microbiol. Rev. 30, 481–502 (2017). 28179378 10.1128/CMR.00090-16 PMC5355638

[26] D. Ren, X. Ye, R. Chen, X. Jia, X. He, J. Tao, T. Jin, S. Wu, H. Zhang, Activation and evasion of inflammasomes during viral and microbial infection. Cell. Mol. Life Sci. 82, 56 (2025). 39833559 10.1007/s00018-025-05575-2 PMC11753444

[27] M. T. T. Htar, M. S. Yerramalla, J. C. Moïsi, D. L. Swerdlow, The burden of respiratory syncytial virus in adults: A systematic review and meta-analysis. Epidem. Infect. 148, e48 (2020). 10.1017/S0950268820000400 PMC7078512 32052719

[28] Z. Teoh, S. Conrey, M. McNeal, A. Burrell, R. M. Burke, C. Mattison, M. McMorrow, D. C. Payne, A. L. Morrow, M. A. Staat, Burden of respiratory viruses in children less than 2 years old in a community-based longitudinal US birth cohort. Clin. Infect. Dis. 77, 901–909 (2023). 37157868 10.1093/cid/ciad289 PMC10838707

[29] A. Agac, S. M. Kolbe, M. Ludlow, A. Osterhaus, R. Meineke, G. F. Rimmelzwaan, Host responses to respiratory syncytial virus infection. Viruses 15, 1999 (2023). 37896776 10.3390/v15101999 PMC10611157

[30] S. O. Vasudevan, B. Behl, V. A. Rathinam, Pyroptosis-induced inflammation and tissue damage. Semin. Immunol. 69, 101781 (2023). 37352727 10.1016/j.smim.2023.101781 PMC10598759

[31] L. Kuo, R. Fearns, P. L. Collins, Analysis of the gene start and gene end signals of human respiratory syncytial virus: Quasi-templated initiation at position 1 of the encoded mRNA. J. Virol. 71, 4944–4953 (1997). 9188557 10.1128/jvi.71.7.4944-4953.1997 PMC191725

[32] C. Griffiths, S. J. Drews, D. J. Marchant, Respiratory syncytial virus: infection, detection, and new options for prevention and treatment. Clin. Microbiol. Rev. 30, 277–319 (2017). 27903593 10.1128/CMR.00010-16 PMC5217795

[33] P. J. M. Openshaw, C. Chiu, F. J. Culley, C. Johansson, Protective and harmful immunity to RSV infection. Annu. Rev. Immunol. 35, 501–532 (2017). 28226227 10.1146/annurev-immunol-051116-052206

[34] L. Lambert, A. M. Sagfors, P. J. Openshaw, F. J. Culley, Immunity to RSV in early-life. Front. Immunol. 5, 466 (2014). 25324843 10.3389/fimmu.2014.00466 PMC4179512

[35] M. K. Tulic, R. J. Hurrelbrink, C. M. Prêle, I. A. Laing, J. W. Upham, P. Le Souef, P. D. Sly, P. G. Holt, TLR4 polymorphisms mediate impaired responses to respiratory syncytial virus and lipopolysaccharide. J. Immunol. 179, 132–140 (2007). 17579031 10.4049/jimmunol.179.1.132

[36] L. Hu, M. Chen, X. Chen, C. Zhao, Z. Fang, H. Wang, H. Dai, Chemotherapy-induced pyroptosis is mediated by BAK/BAX-caspase-3-GSDME pathway and inhibited by 2-bromopalmitate. Cell Death Dis. 11, 281 (2020). 32332857 10.1038/s41419-020-2476-2 PMC7181755

[37] R. Heilig, M. Dilucca, D. Boucher, K. W. Chen, D. Hancz, B. Demarco, K. Shkarina, P. Broz, Caspase-1 cleaves bid to release mitochondrial SMAC and drive secondary necrosis in the absence of GSDMD. Life Sci. Alliance 3, e202000735 (2020). 32345661 10.26508/lsa.202000735 PMC7190276

[38] D. Milhas, O. Cuvillier, N. Therville, P. Clavé, M. Thomsen, T. Levade, H. Benoist, B. Ségui, Caspase-10 triggers Bid cleavage and caspase cascade activation in FasL-induced apoptosis. J. Biol. Chem. 280, 19836–19842 (2005). 15772077 10.1074/jbc.M414358200

[39] M. Brentnall, L. Rodriguez-Menocal, R. L. De Guevara, E. Cepero, L. H. Boise, Caspase-9, caspase-3 and caspase-7 have distinct roles during intrinsic apoptosis. BMC Cell Biol. 14, 32 (2013). 23834359 10.1186/1471-2121-14-32 PMC3710246

[40] A. Balasubramanian, A. Y. Hsu, L. Ghimire, M. Tahir, P. Devant, P. Fontana, G. Du, X. Liu, D. Fabin, H. Kambara, X. Xie, F. Liu, T. Hasegawa, R. Xu, H. Yu, M. Chen, S. Kolakowski, S. Trauger, M. R. Larsen, W. Wei, H. Wu, J. C. Kagan, J. Lieberman, H. R. Luo, The palmitoylation of gasdermin D directs its membrane translocation and pore formation during pyroptosis. Sci. Immunol. 9, eadn1452 (2024). 38530158 10.1126/sciimmunol.adn1452 PMC11367861

[41] G. Du, L. B. Healy, L. David, C. Walker, T. J. El-Baba, C. A. Lutomski, B. Goh, B. Gu, X. Pi, P. Devant, P. Fontana, Y. Dong, X. Ma, R. Miao, A. Balasubramanian, R. Puthenveetil, A. Banerjee, H. R. Luo, J. C. Kagan, S. F. Oh, C. V. Robinson, J. Lieberman, H. Wu, ROS-dependent S-palmitoylation activates cleaved and intact gasdermin D. Nature 630, 437–446 (2024). 38599239 10.1038/s41586-024-07373-5 PMC11283288

[42] M. Li, J. Li, R. Zeng, J. Yang, J. Liu, Z. Zhang, X. Song, Z. Yao, C. Ma, W. Li, K. Wang, L. Wei, Respiratory syncytial virus replication is promoted by autophagy-mediated inhibition of apoptosis. J. Virol. 92, (2018), 10.1128/JVI.02193-17. PMC5874425 29386287

[43] K. Chiok, S. M. Pokharel, I. Mohanty, L. G. Miller, S. J. Gao, A. L. Haas, K. C. Tran, M. N. Teng, S. Bose, human respiratory syncytial virus NS2 protein induces autophagy by modulating beclin1 protein stabilization and ISGylation. MBio 13, e0352821 (2022). 35038909 10.1128/mbio.03528-21 PMC8764521

[44] R. Medzhitov, The spectrum of inflammatory responses. Science 374, 1070–1075 (2021). 34822279 10.1126/science.abi5200

[45] G. van Loo, M. J. M. Bertrand, Death by TNF: A road to inflammation. Nat. Rev. Immunol. 23, 289–303 (2023). 36380021 10.1038/s41577-022-00792-3 PMC9665039

[46] P. R. Vajjhala, A. Lu, D. L. Brown, S. W. Pang, V. Sagulenko, D. P. Sester, S. O. Cridland, J. M. Hill, K. Schroder, J. L. Stow, H. Wu, K. J. Stacey, The inflammasome adaptor ASC induces procaspase-8 death effector domain filaments. J. Biol. Chem. 290, 29217–29230 (2015). 26468282 10.1074/jbc.M115.687731 PMC4705927

[47] M. A. Horga, S. Macip, A. C. Tuyama, M. C. Tan, G. L. Gusella, Human parainfluenza virus 3 neuraminidase activity contributes to dendritic cell maturation. Viral Immunol. 18, 523–533 (2005). 16212531 10.1089/vim.2005.18.523

[48] H. Plotnicky-Gilquin, D. Cyblat, J. P. Aubry, Y. Delneste, A. Blaecke, J. Y. Bonnefoy, N. Corvaïa, P. Jeannin, Differential effects of parainfluenza virus type 3 on human monocytes and dendritic cells. Virology 285, 82–90 (2001). 11414808 10.1006/viro.2001.0933

[49] Z. T. Schug, F. Gonzalvez, R. H. Houtkooper, F. M. Vaz, E. Gottlieb, BID is cleaved by caspase-8 within a native complex on the mitochondrial membrane. Cell Death Differ. 18, 538–548 (2011). 21072056 10.1038/cdd.2010.135 PMC3132005

[50] P. Smyth, T. Sessler, C. J. Scott, D. B. Longley, FLIP(L): The pseudo-caspase. FEBS J. 287, 4246–4260 (2020). 32096279 10.1111/febs.15260 PMC7586951

[51] Y. C. Kang, K. M. Kim, K. S. Lee, S. Namkoong, S. J. Lee, J. A. Han, D. Jeoung, K. S. Ha, Y. G. Kwon, Y. M. Kim, Serum bioactive lysophospholipids prevent TRAIL-induced apoptosis via PI3K/Akt-dependent cFLIP expression and Bad phosphorylation. Cell Death Differ. 11, 1287–1298 (2004). 15297884 10.1038/sj.cdd.4401489

[52] K. W. Thomas, M. M. Monick, J. M. Staber, T. Yarovinsky, A. B. Carter, G. W. Hunninghake, Respiratory syncytial virus inhibits apoptosis and induces NF-κB activity through a phosphatidylinositol 3-kinase-dependent pathway. J. Biol. Chem. 277, 492–501 (2002). 11687577 10.1074/jbc.M108107200

[53] R. O’Donnell, L. Milligan, J. M. Stark, Induction of CD95 (Fas) and apoptosis in respiratory epithelial cell cultures following respiratory syncytial virus infection. Virology 257, 198–207 (1999). 10208933 10.1006/viro.1999.9650

[54] A. Leemans, M. Boeren, W. Van der Gucht, W. Martinet, G. Caljon, L. Maes, P. Cos, P. Delputte, Characterization of the role of N-glycosylation sites in the respiratory syncytial virus fusion protein in virus replication, syncytium formation and antigenicity. Virus Res. 266, 58–68 (2019). 31004621 10.1016/j.virusres.2019.04.006

[55] C. A. Malinczak, C. F. Schuler, A. J. Duran, A. J. Rasky, M. M. Mire, G. Núñez, N. W. Lukacs, W. Fonseca, NLRP3-inflammasome inhibition during respiratory virus infection abrogates lung immunopathology and long-term airway disease development. Viruses 13, 692 (2021). 33923693 10.3390/v13040692 PMC8072578

[56] C. Shen, Z. Zhang, T. Xie, J. Ji, J. Xu, L. Lin, J. Yan, A. Kang, Q. Dai, Y. Dong, J. Shan, S. Wang, X. Zhao, Rhein suppresses lung inflammatory injury induced by human respiratory syncytial virus through inhibiting NLRP3 inflammasome activation via NF-κB pathway in mice. Front. Pharmacol. 10, 1600 (2020). 32047436 10.3389/fphar.2019.01600 PMC6997271

[57] P. Klein Klouwenberg, L. Tan, W. Werkman, G. M. van Bleek, F. Coenjaerts, The role of Toll-like receptors in regulating the immune response against respiratory syncytial virus. Crit. Rev. Immunol. 29, 531–550 (2009). 20121698 10.1615/critrevimmunol.v29.i6.40

[58] H. I. Muendlein, W. M. Connolly, J. Cameron, D. Jetton, Z. Magri, I. Smirnova, E. Vannier, X. Li, A. J. Martinot, R. Batorsky, A. Poltorak, Neutrophils and macrophages drive TNF-induced lethality via TRIF/CD14-mediated responses. Sci. Immunol. 7, eadd0665 (2022). 36563168 10.1126/sciimmunol.add0665 PMC10021564

[59] S. Liu, L. Gao, X. Wang, Y. Xing, Respiratory syncytial virus infection inhibits TLR4 signaling via up-regulation of miR-26b. Cell Biol. Int. 39, 1376–1383 (2015). 26222045 10.1002/cbin.10518

[60] S. G. Verburg, R. M. Lelievre, M. J. Westerveld, J. M. Inkol, Y. L. Sun, S. T. Workenhe, Viral-mediated activation and inhibition of programmed cell death. PLOS Pathog. 18, e1010718 (2022). 35951530 10.1371/journal.ppat.1010718 PMC9371342

[61] C. D. Griffiths, L. M. Bilawchuk, J. E. McDonough, K. C. Jamieson, F. Elawar, Y. Cen, W. Duan, C. Lin, H. Song, J. L. Casanova, S. Ogg, L. D. Jensen, B. Thienpont, A. Kumar, T. C. Hobman, D. Proud, T. J. Moraes, D. J. Marchant, IGF1R is an entry receptor for respiratory syncytial virus. Nature 583, 615–619 (2020). 32494007 10.1038/s41586-020-2369-7

