# Supplementary material for: RSV temporally reprograms apoptosis and pyroptosis to balance immune evasion and replication
Source: Sci Adv. 2026 Jan 23;12(4):eadz2496. doi: 10.1126/sciadv.adz2496 (PMC12829576; doi:10.1126/sciadv.adz2496)
Supplement: Supplementary file 1 — Figs. S1 to S10 Tables S1 to S3 [file sciadv.adz2496_sm.pdf]

Science Advances
AAAS

Supplementary Materials for

RSV temporally reprograms apoptosis and pyroptosis to balance immune evasion and replication

Cong Liu *et al.*

Corresponding author: Mingzhou Chen, chenmz@hubu.edu.cn; Yali Qin, yqin@hubu.edu.cn

Sci. Adv. **12**, eadz2496 (2026)
DOI: 10.1126/sciadv.adz2496

This PDF file includes:

Figs. S1 to S10
Tables S1 to S3

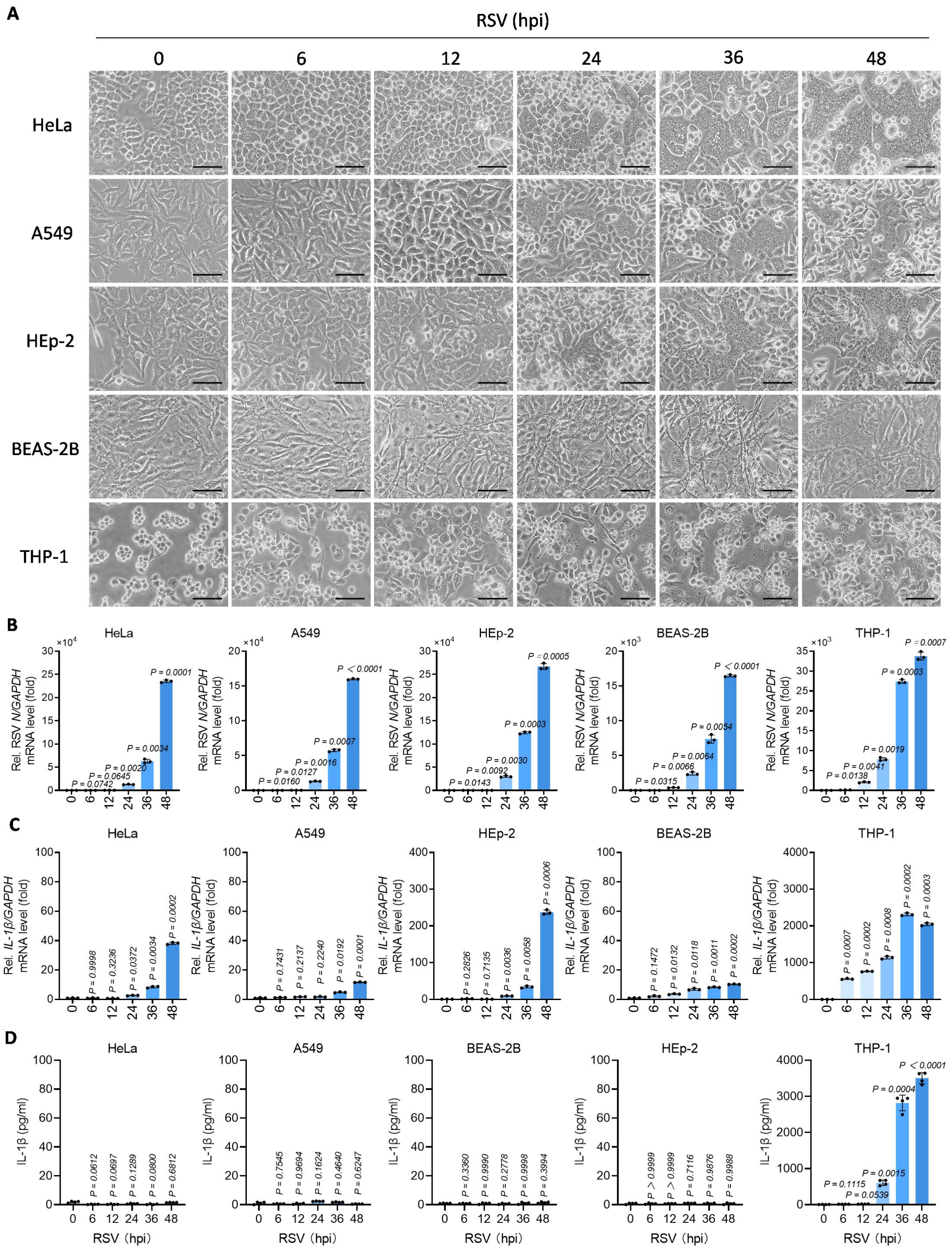

Fig. S1. RSV triggers cytopathology and modulates IL-1β biosynthesis and secretion in diverse immune and non-immune cells. (**A** to **D**) HeLa, A549, HEp-2, BEAS-2B and PMA-differentiated THP-1 macrophages were infected with RSV (MOI=1.0) for indicated times.

Cytomorphological changes under microscopy (A). Scale bar, 100 μM. The intracellular mRNA levels of RSV N (B) or IL-1β (C) were measured by qRT-PCR, and the level of mRNA in mock cells were defined as 1-fold. Supernatants were collected for detection of IL-1β secretion by ELISA (D). Graphs show mean ± s.d (n=3 biologically independent experiments). Statistical significance was determined by two way ANOVA in (A and C). hpi, hours post-infection.

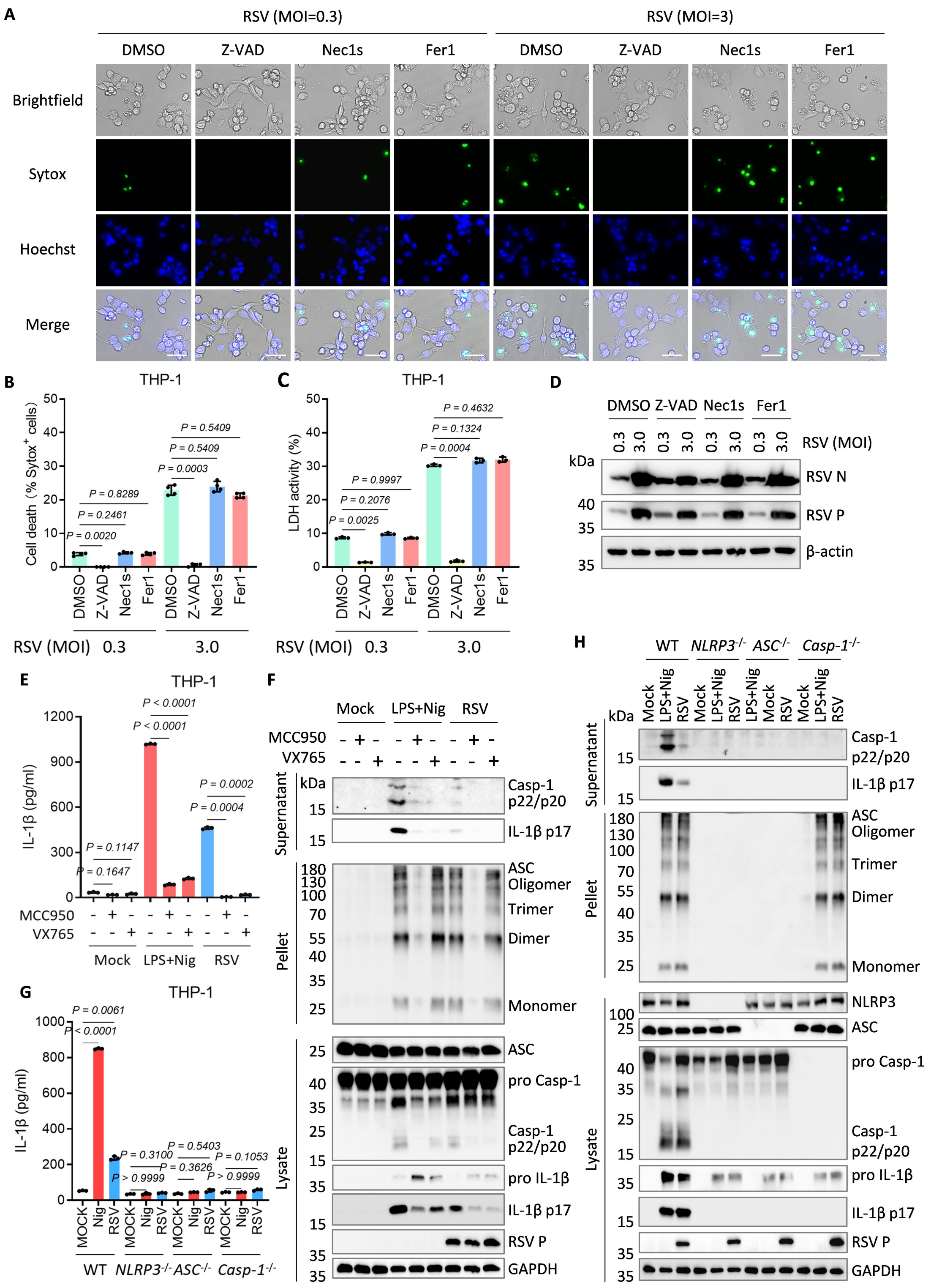

Fig. S2. RSV-induced cell death and IL-1β release depend on NLRP3 and caspase activition. (**A**) Cell death in PMA-differentiated THP-1 cells using Sytox Green and Hoechst staining after infection with RSV (MOI=0.3 or 3.0) for 24 hours in the presence/absence of the pan-caspase

inhibitor Z-VAD-FMK (Z-VAD, 30 μM), Necroptosis inhibitor Necrostatin-1s (Nec1s, 30 μM), or the Ferroptosis inhibitor Ferrostatin-1 (Fer1, 10 μM). The green or blue denote the dead cells or all cells counted during the analysis. Scale bar, 50 μM. (**B** to **D**) Quantification of the cell death (B), LDH release in supernatants (C) and virus proteins expression in lysates (D) in (A). (**E** and **F**) PMA-differentiated THP-1 macrophages were infected with RSV (MOI=3.0) for 24 hours or treated with LPS (1 μg/ml) for 4 hours plus Nigericin (2.5 μM) (LPS+Nig) for 2 hours in the presence/absence of the NLRP3 inhibitor MCC950 (20 μM) or casapse-1 inhibitor VX765 (50 μM). Supernatants were collected for detection of IL-1β secretion by ELISA (E). Immunoblots of the indicated proteins in the supernatants, pellets and lysates (F). (**G** and **H**) PMA-differentiated WT or genetic knockout THP-1 macrophages were infected with RSV (MOI=3.0) for 24 hours or treated with LPS (1 μg/ml) for 4 hours plus Nigericin (2.5 μM) (LPS+Nig) for 2 hours. Supernatants were collected for detection of IL-1β secretion by ELISA (G). Immunoblots of the indicated proteins in the supernatants, pellets and lysates (H). Graphs show mean ± s.d (n=3 biologically independent experiments). Statistical significance was determined by two way ANOVA in (B, C, E and G). hpi, hours post-infection. Casp, Caspase.

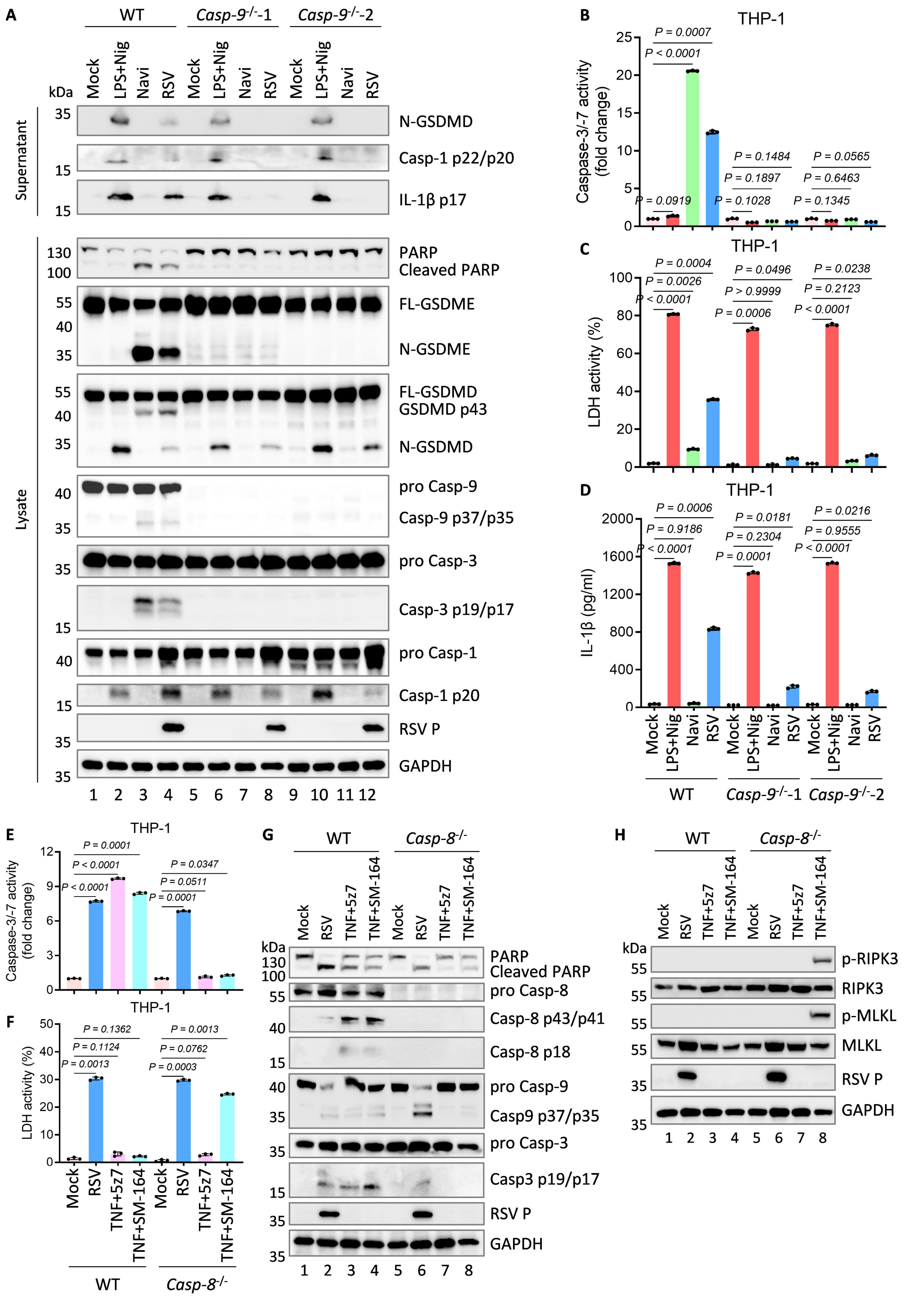

Fig. S3. RSV-induced pyroptosis and IL-1β release are dependent on caspase-9 but not caspase-8. (**A** to **D**) PMA-differentiated WT or two *Casp-9*$^{-/-}$ (*Casp-9*$^{-/-}$-1 and -2) THP-1

macrophages were infected with RSV (MOI=3.0) for 24 hours. Cell supernatants and lysates and were analyzed by immunoblots (A), caspase-3/-7 activity assay (B), LDH release detection (C) and IL-1β ELISA (D). (**E** to **H**) PMA-differentiated WT or *Casp*-$8^{-/-}$ THP-1 macrophages were infected with RSV (MOI=3.0) for 24 hours or treated with TNF (20 ng/mL) plus 5z7 (5z-7-oxozeaenol, 4μM) for 5 hours or TNF (100 ng/mL) plus SM-164 (1 μM) for 12 hours. Cell lysates and pellets were analyzed by caspase-3/-7 activity assay (E) and immunoblots (G and H). Supernatants were collected and subjected to measurements of LDH release (F). Graphs show mean ± s.d (n=3 biologically independent experiments). Statistical significance was determined by two way ANOVA in (B to F). Casp, Caspase. FL-GSDMD, Full length GSDMD. N-GSDMD, N-terminal GSDMD. FL-GSDME, Full length GSDME. N-GSDME, N-terminal GSDME.

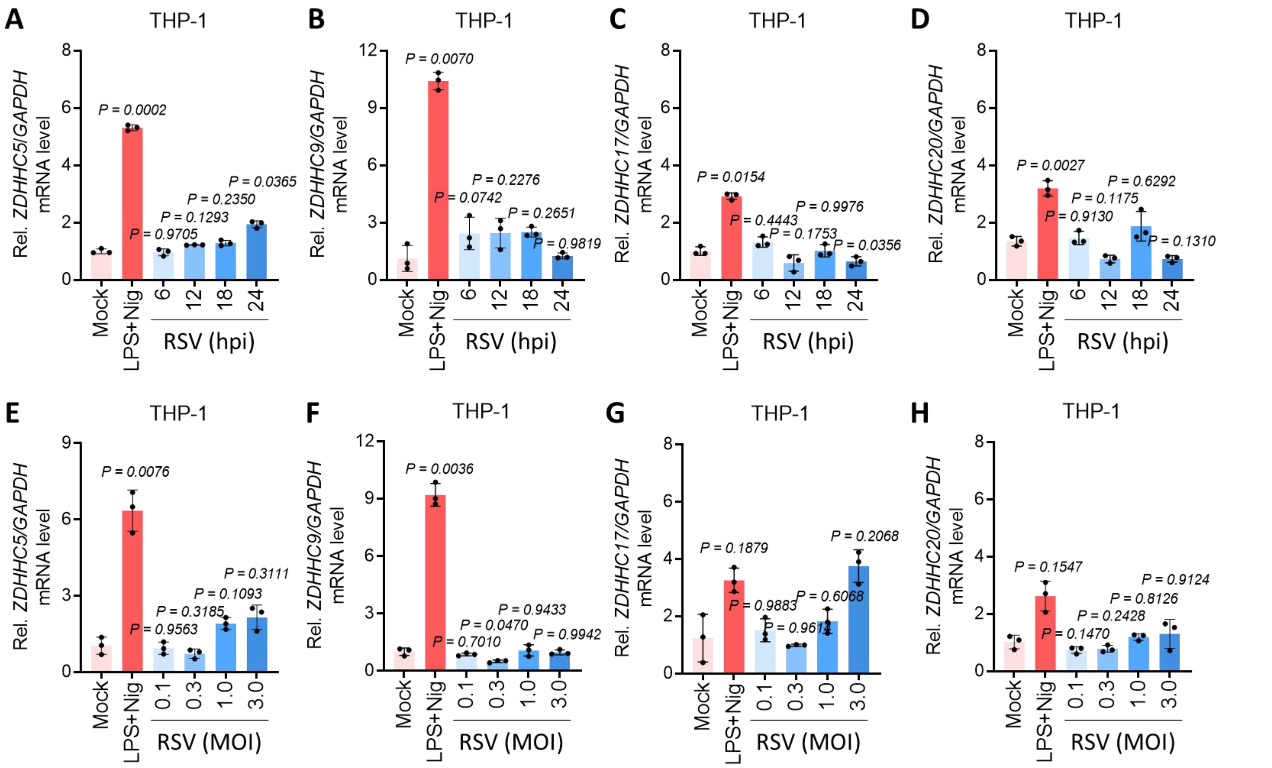

Fig. S4. RSV has no effect on the mRNA levels of ZDHHC9. (**A** to **H**) PMA-differentiated *Casp-9*$^{-/-}$ THP-1 macrophages were infected with RSV (MOI=3.0) for indicated times (A to D) or at different MOI for 12 hours (E to H), with LPS (1 µg/mL, 4 hours) plus Nigericin (2.5 µM, 2 hour) as the positive control of pyroptosis. RT-qPCR was performed to measure the transcription of indicated genes. Graphs show mean ± s.d (n=3 biologically independent experiments). Statistical significance was determined by one way ANOVA in (A to H). hpi, hours post-infection.

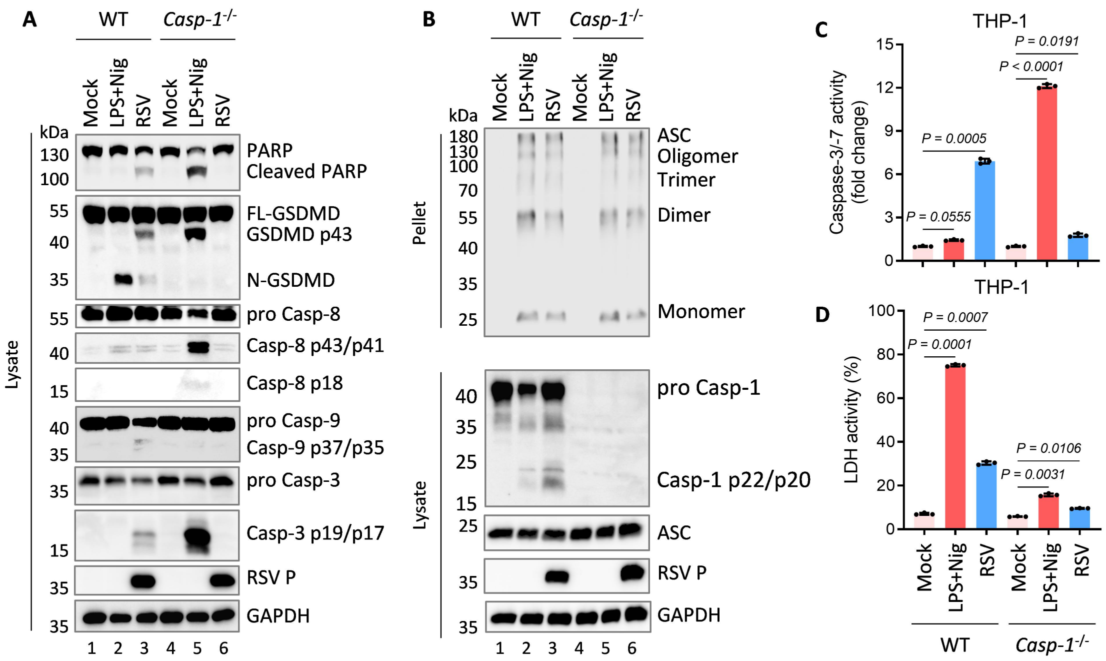

Fig. S5. RSV inhibits apoptosis driven by ASC following the activation of NLRP3 in caspase-1-deficient macrophages. (**A** to **D**) PMA-differentiated WT or *Casp-1*$^{-/-}$ THP-1 macrophages were infected with RSV (MOI=3.0) for 24 hours or treated with LPS (1 μg/mL) for 4 hours plus Nigericin (Nig, 2.5 μM) 2 hours. Cell lysates and pellets were analyzed by immunoblots (A and B), or caspase-3/-7 activity assay (C). Cell supernatant were collected and subjected to measurements of LDH release (D). Graphs show mean ± s.d (n=3 biologically independent experiments). Statistical significance was determined by two way ANOVA in (C and D). Casp, Caspase. FL-GSDMD, Full length GSDMD. N-GSDMD, N-terminal GSDMD.

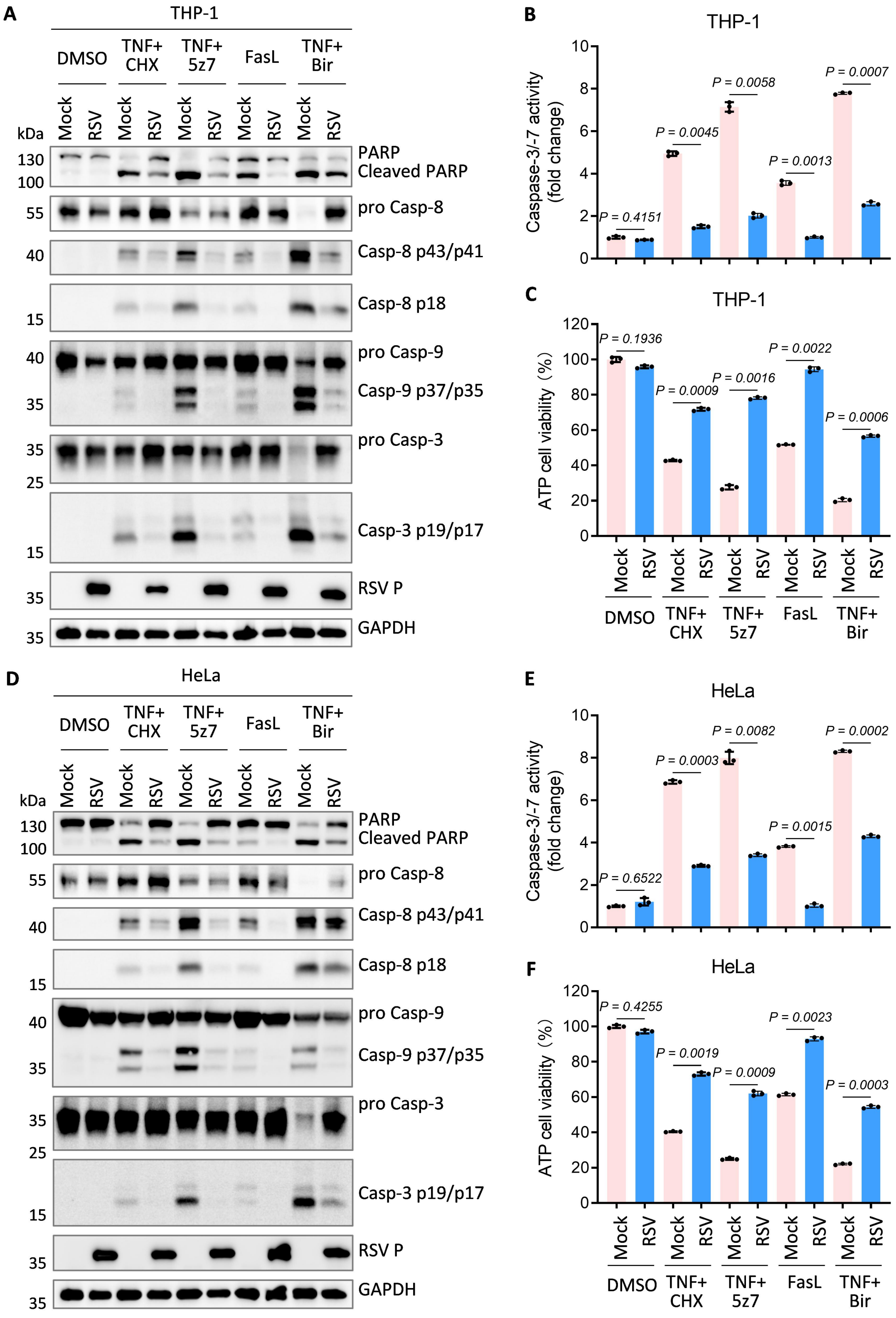

Fig. S6. RSV blocks apoptosis induced by various extrinsic apoptosis inducers in both THP-1 and HeLa cells. (**A** to **C**) PMA-differentiated THP-1 macrophages were infected with

RSV (MOI=3.0) for 6-8 hours and than stimulated with TNF (20 ng/mL) plus CHX (Cycloheximide, 10 μg/mL) for 4 hours, TNF (20 ng/mL) plus 5z7 (5z-7-oxozeaenol, 4 μM) for 5 hours, FasL (200 ng/mL) for 6 hours, or TNF (20 ng/mL) plus Bir (Birinapant, 20 ng/mL) for 4 hours. Cell lysates were analyzed by immunoblots (A), caspase-3/-7 activity assay (B), or cell viability (ATP Glo) evaluation (C). (**D** to **F**) WT HeLa cells were infected with RSV (MOI=3.0) for 6-8 hours and than stimulated with TNF (20 ng/mL) plus CHX (Cycloheximide, 10 μg/mL) for 4 hours, TNF (20 ng/mL) plus 5z7 (5z-7-oxozeaenol, 4 μM) for 5 hours, FasL (200 ng/mL) for 6 hours, or TNF (20 ng/mL) plus Bir (Birinapant, 20 ng/mL) for 4 hours. Cell lysates were analyzed by immunoblots (D), caspase-3/-7 activity assay (E), or cell viability (ATP Glo) evaluation (F). Graphs show mean ± s.d (n=3 biologically independent experiments). Statistical significance was determined by two way ANOVA in (B, C, E, and F). Casp, Caspase.

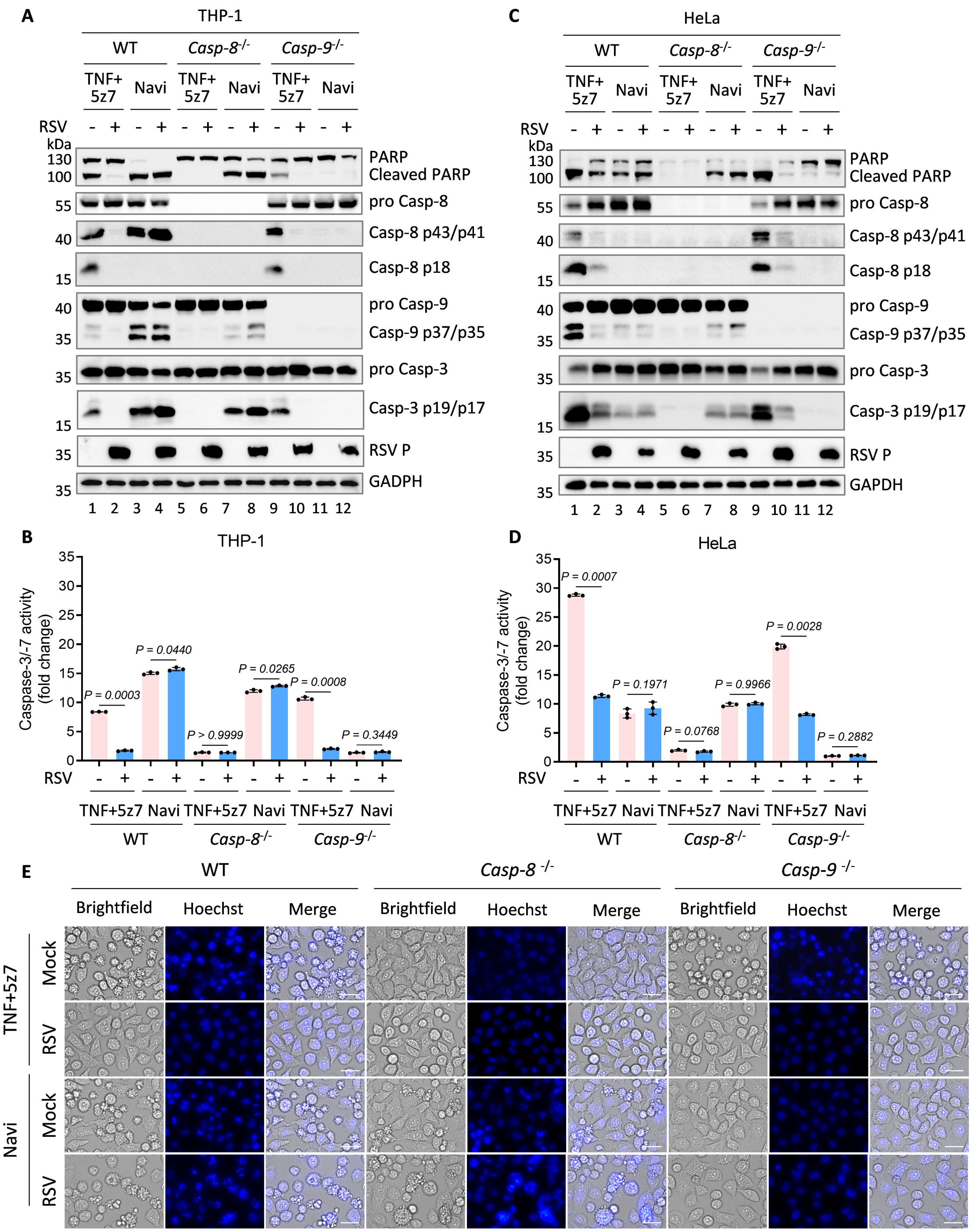

Fig. S7. RSV inhibits TNF-induced cleavage of caspase-9 in a caspase-8-dependent manner in both THP-1 and HeLa cells. (**A** and **B**) PMA-differentiated WT or genetic knockout THP-1 macrophages were infected with RSV (MOI=3.0) for 7 hours and than stimulated with TNF (20 ng/mL) plus 5z7 (5z-7-oxozeaenol, 4µM) or Navi (Navitoclax, 50 µM) for 5 hours. Cell lysates were analyzed by immunoblots (A) or caspase-3/-7 activity assay (B). (**C** and **D**) WT or genetic

knockout HeLa cells were infected with RSV (MOI=3.0) for 7 hours and than stimulated with TNF (20 ng/mL) plus 5z7 (5z-7-oxozeaenol, 4μM) or Navi (Navitoclax, 50 μM) for 5 hours. Cell lysates were analyzed by immunoblots (C) or caspase-3/-7 activity assay (D). (**E**) Hoechst stain of WT, *Casp-8*$^{-/-}$ or *Casp-9*$^{-/-}$ HeLa cells after infection with RSV (MOI=3.0) for 7 hours and than stimulated with TNF (20 ng/mL) plus 5z7 (5z-7-oxozeaenol, 4 μM) or Navi (Navitoclax, 50 μM) for 5 hours. The blue denotes the nucleus. Scale bar, 50 μM. Graphs show mean ± s.d (n=3 biologically independent experiments). Statistical significance was determined by two way ANOVA in (B). Casp, Caspase.

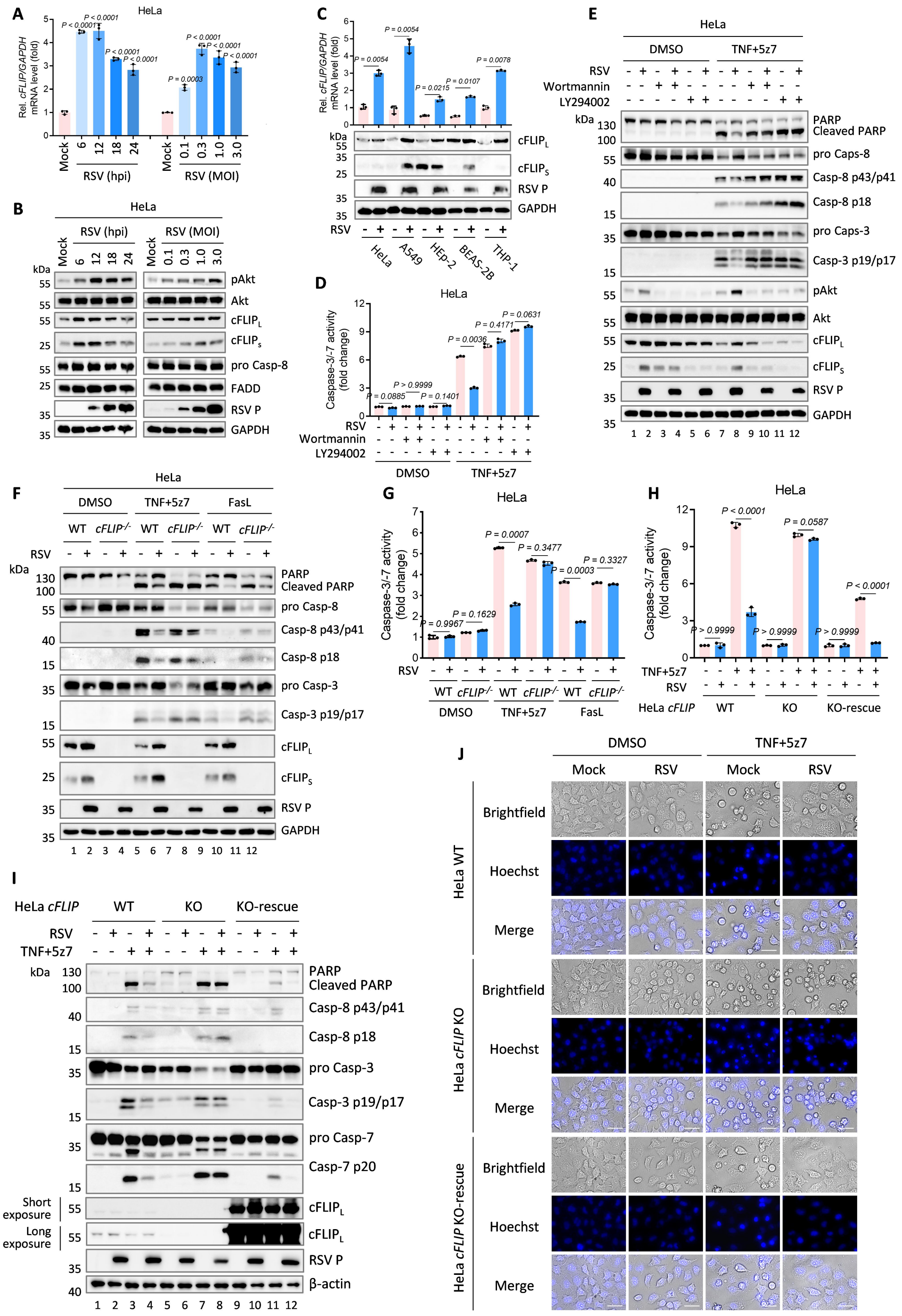

Fig. S8. cFLIP is involved in the inhibition of RSV-induced extrinsic apoptosis in non-immune cells. (**A** and **B**) HeLa cells were infected with RSV (MOI=3.0) for indicated times or

at different MOI for 12 hours. Cells were collected and RT-qPCR was performed to measure the mRNA levels of indicated genes (A). Cell lysates were analyzed by immunoblots (B). (**C**) HeLa, A549, HEp-2, BEAS-2B and THP-1 cells were infected with RSV (MOI=3.0) for 12 hours. Cells were collected and RT-qPCR was performed to measure the mRNA levels of indicated genes (above). Cell lysates were analyzed by immunoblots (below). (**D** and **E**) HeLa cells were infected with RSV (MOI=3.0) for 7 hours in the presence/absence of Wortmannin (200 μM) or LY294002 (10 μM), and than stimulated with TNF (20 ng/mL) plus 5z7 (5z-7-oxozeaenol, 4 μM) for 5 hours. Cell lysates were analyzed by caspase-3/-7 activity assay (D) or immunoblots (E). (**F** and **G**) WT or *cFLIP*$^{-/-}$ HeLa cells were infected with RSV (MOI=3.0) for 6-7 hours, and than stimulated with TNF (20 ng/mL) plus 5z7 (5z-7-oxozeaenol, 4 μM) for 5 hours or FasL (200 ng/mL) for 6 hours. Cell lysates were analyzed by by immunoblots (F) or caspase-3/-7 activity assay (G). (**H** to **I**) WT, *cFLIP* KO or *cFLIP* KO-rescue HeLa cells were infected with RSV (MOI=3.0) for 7 hours, and than stimulated with TNF (20 ng/mL) plus 5z7 (5z-7-oxozeaenol, 4 μM) for 5 hours. Cell lysates were analyzed by by caspase-3/-7 activity assay (H) or immunoblots (I). (**J**) Hoechst stain of WT, *cFLIP* KO or *cFLIP* KO-rescue HeLa cells after infection with RSV (MOI= 3.0) for 7 hours and than stimulation with TNF (20 ng/mL) plus 5z7 (5z-7-oxozeaenol, 4 μM) for 5 hours. The blue denotes the nucleus. Scale bar, 50 μM. Graphs show mean ± s.d (n=3 biologically independent experiments). Statistical significance was determined by two way ANOVA in (A, C, D, G, and H). Casp, Caspase. hpi, hours post-infection.

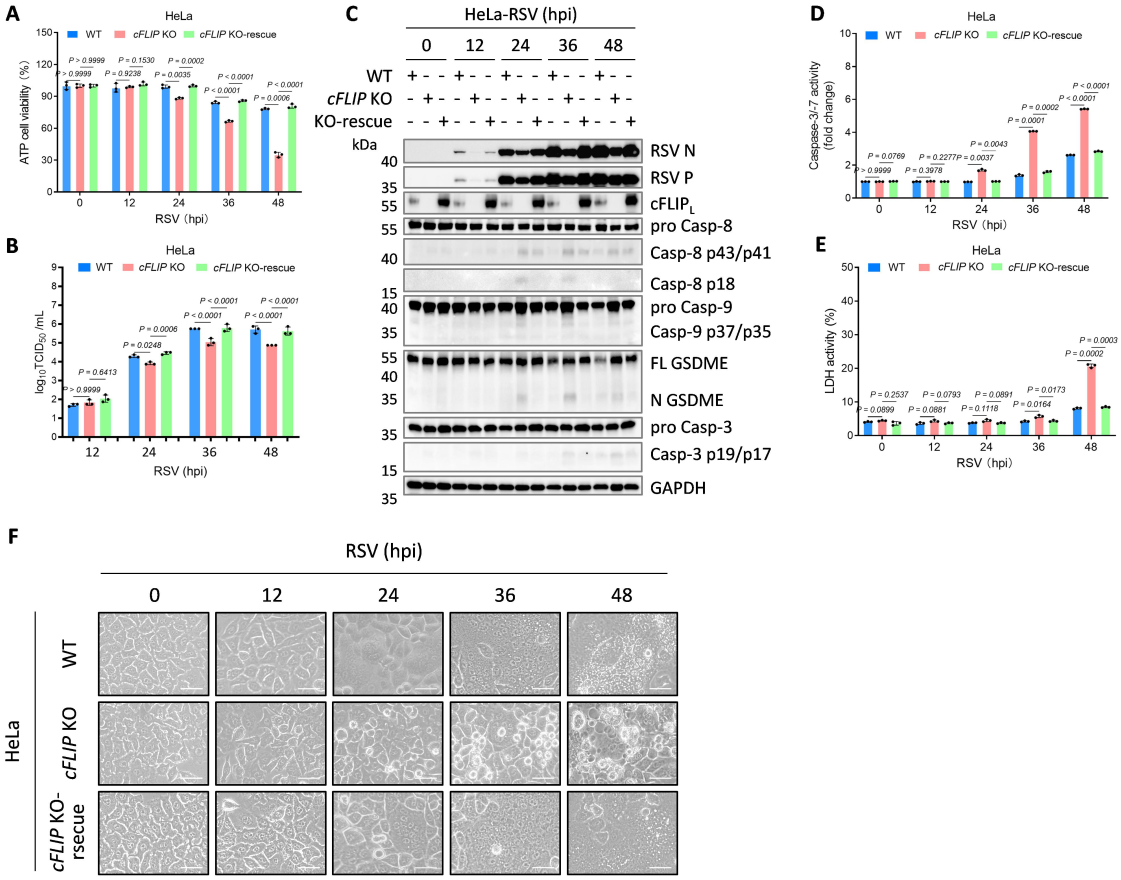

Fig. S9. RSV exploits cFLIP to facilitate virus replication and syncytium formation in HeLa cells. (**A** to **F**), WT, *cFLIP* KO or *cFLIP* KO-rescue HeLa cells were infected with RSV (MOI=3.0) for indicated times. The percentage of viable cells was determined through cell viability (ATP Glo) evaluation (A). Cell lysates were analyzed by immunoblots (C) and caspase-3/-7 activity assay (D), and supernatants were harvested for measurements of the viral titers with standard $TCID_{50}$ assays (B) and LDH release (E). Cytomorphological changes under microscopy (F). Scale bar, 50 μM. Graphs show mean ± s.d (n=3 biologically independent experiments). Statistical significance was determined by two way ANOVA in (A, B, D, and E). Casp, Caspase. FL-GSDME, Full length GSDME. N-GSDME, N-terminal GSDME. hpi, hours post-infection.

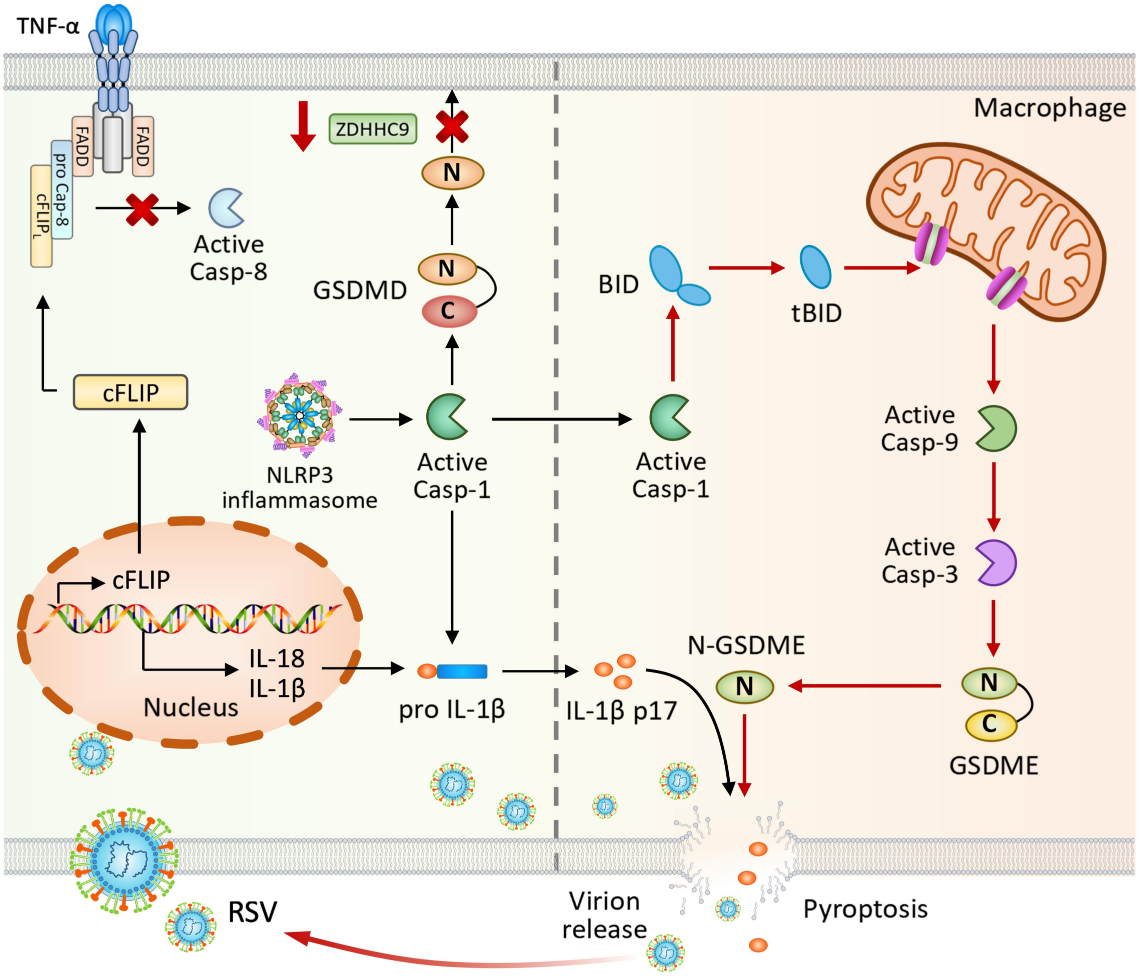

Fig. S10. Model of programmed cell death regulated by RSV for viral replication.

Following infection of THP-1 macrophages, RSV employs a biphasic strategy: Early infection involves cFLIP upregulation to block TNF-induced extrinsic apoptosis, coupled with ZDHHC9 degradation to inhibit N-GSDMD-mediated pyroptosis after NLRP3 inflammation activation; Post-replication, RSV redirects caspase-1 signals to initiate intrinsic apoptosis via the Casp-1-BID-APAF1-Casp-9 axis, ultimately inducing N-GSDME-dependent pyroptosis. This facilitates the massive release of viral particles and inflammatory cytokines. Casp, Caspase

Table S1. Plasmids and oligonucleotides

Name	Description	Source
**Plasmids**		
pSPAX2	Used to package lentiviruses with pMD2.G and lentiCRISPRv2	Addgene (Cat# 12260)
pMD2.G	Used to package lentiviruses with pSPAX2 and lentiCRISPRv2	Addgene (Cat# 12259)
lentiCRISPRv2	Lentiviral transfer plasmid used to create puromycin resistance sgRNA for gene knockout	Addgene (Cat# 52961)
pCDH-CMV-IRES-MCS-SF-BLAST	Lentiviral transfer plasmid used to create blasticidin resistance gene for stable expression	Dong Guo, et al. 2022
pCDH-CMV-cFLIP Mut	For stable expression of cFLIP	This study
**Oligonucleotides**	**(5'-3')**	
cFLIP-NheI-F	CACGCTAGCATGTCTGCTGAAGTCAT	This study
cFLIP-NotI-R	CACGCGGCCGCTGTGTAGGAGAGGATAA	This study
cFLIP Mut-F	GGACGTGGTAAAATCACCAAAGAGAAGAGTTT	This study
cFLIP Mut-R	TTCTCTTTGGTGATTTTACCACGTCCCATGTAAT	This study
pCDH-cFLIP-F	TCAGATCTCGAGATGTCTGCTGAAGTCATC	This study
pCDN-cFLIP-R	CTCCAGGATCCTGTGTAGGAGAGGATAAGTTT	This study
pCDH-F	TCTCCTACACAGGATCCTGGAGCCACCCTC	This study
pCDH-R	TTCAGCAGACATCTCGAGATCTGAGTCCGG	This study
sgGSDMD-1-F	CACCGACAGGCAAAGATCGCAGG	This study
sgGSDMD-1-R	AAACCCTGCGATCTTTGCCTGTC	This study
sgGSDMD-2-F	CACCGCGCGCACCCACAAGCGGGA	This study
sgGSDMD-2-R	AAACTCCCGCTTGTGGGTGCGCGC	This study
sgGSDME-1-F	CACCGTATAACTCAATGACACCGT	This study
sgGSDME-1-R	AAACACGGTGTCATTGAGTTATAC	This study
sgGSDME-2-F	CACCGGGATCTCCAGCACCACGT	This study
sgGSDME-2-R	AAACACGTGGTGCTGGAGATCCC	This study
sgASC-1-F	CACCGTTGGACCTCACCGACAAGC	This study
sgASC-1-R	AAACGCTTGTCGGTGAGGTCCAAC	This study
sgASC-2-F	CACCGAACTTCTTGAGCTCCTCGG	This study
sgASC-2-R	AAACCCGAGGAGCTCAAGAAGTTC	This study
sgCasp-1-1-F	CACCGACAGTATTCCTAGAAGAAC	This study
sgCasp-1-1-R	AAACGTTCTTCTAGGAATACTGTC	This study
sgCasp-1-2-F	CACCGCTCCCTAGAAGAAGCTCAA	This study
sgCasp-1-2-R	AAACTTGAGCTTCTTCTAGGGAGC	This study
sgCasp8-1-F	CACCGCCTGGACTACATTCCGCAA	This study
sgCasp8-1-R	AAACTTGCGGAATGTAGTCCAGGC	This study
sgCasp8-2-F	CACCGAATTAATAGACTGGATTTG	This study
sgCasp8-2-R	AAACCAAATCCAGTCTATTAATTC	This study
sgCasp9-1-F	CACCGCATCGACTGTGAGAAGTTG	This study
sgCasp9-1-R	AAACCAACTTCTCACAGTCGATGC	This study
sgCasp9-2-F	CACCGTGTCGGTCGAGAAGATTG	This study
sgCasp9-2-R	AAACCAATCTTCTCGACCGACAC	This study
sgNLRP3-1-F	CACCGAGGACTATCCTCCCCAGAA	This study
sgNLRP3-1-R	AAACTTCTGGGGAGGATAGTCCTC	This study

**Continued**		
**Oligonucleotides**	**(5'-3')**	
sgNLRP3-2-F	CACCGCTGCAAGCTGGCCAGGTACC	This study
sgNLRP3-2-R	AAACGGTACCTGGCCAGCTTGCAGC	This study
sgFLIP-1-F	CACCGGCCGAGGCAAGATAAGCA	This study
sgFLIP-1-R	AAACTGCTTATCTTGCCTCGGCC	This study
sgFLIP-2-F	CACCGCACAAAGCTGTCGTAGTCT	This study
sgFLIP-2-R	AAACAGACTACGACAGCTTTGTGC	This study
sgBID-1-F	CACCGCTCAGGAACACCAGCCGGT	This study
sgBID-1-R	AAACACCGGCTGGTGTTCCTGAGC	This study
sgBID-2-F	CACCGAACCTACGCACCTACGTG	This study
sgBID-2-R	AAACCACGTAGGTGCGTAGGTTC	This study
sgAPAF1-1-F	CACCGTGGGTCACCATACATGGAA	This study
sgAPAF1-1-R	AAACTTCCATGTATGGTGACCCAC	This study
sgAPAF1-2-F	CACCGAGTGCATTGGGTTTCAGTT	This study
sgAPAF1-2-R	AAACAACTGAAACCCAATGCACTC	This study

Table S2. Antibodies

Antibodes	Source	Identifier
Mouse monoclonal anti-IL-1β	Cell Signaling Technology	Cat# 12242
Rabbit monoclonal anti-Cleaved-IL-1β	Cell Signaling Technology	Cat# 83186
Mouse monoclonal anti-Caspase-1	Adipogen Life Sciences	Cat# AG-20B-0048-C100
Mouse monoclonal anti-ASC	Santa Cruz Biotechnology	Cat# sc-514414
Rabbit monoclonal anti-NLRP3	Cell Signaling Technology	Cat# 13158
Rabbit monoclonal anti-Gasdermin D	Cell Signaling Technology	Cat# 97558
Rabbit monoclonal anti-GSDME	Abcam	Cat# ab215191
Rabbit polyclonal anti-Caspase-3	Cell Signaling Technology	Cat# 9662
Rabbit monoclonal anti-Cleaved Caspase-3	Cell Signaling Technology	Cat# 9664
Mouse monoclonal anti-Caspase-7	Cell Signaling Technology	Cat# 9494
Rabbit monoclonal anti-Caspase-8	Cell Signaling Technology	Cat# 4790
Rabbit monoclonal anti-Cleaved Caspase-8	Cell Signaling Technology	Cat# 98134
Mouse monoclonal anti-Caspase-9	Cell Signaling Technology	Cat# 9508
Rabbit polyclonal anti-Caspase-10	Abcepta	Cat# AP63182
Rabbit polyclonal anti-PARP	Cell Signaling Technology	Cat# 9542
Rabbit polyclonal anti-BID	Cell Signaling Technology	Cat# 2002
Rabbit monoclonal anti-APAF1	Cell Signaling Technology	Cat# 8723
Rabbit monoclonal anti-Phospho-Akt (Ser473)	Cell Signaling Technology	Cat# 4060
Rabbit monoclonal anti-Akt (pan)	Cell Signaling Technology	Cat# 4691
Rabbit monoclonal anti-RIPK3	Cell Signaling Technology	Cat# 10188S
Rabbit monoclonal anti-Phospho-RIP3	Cell Signaling Technology	Cat# 93654S
Rabbit monoclonal anti-MLKL	Cell Signaling Technology	Cat# 14993S
Rabbit monoclonal anti-Phospho-MLKL	Cell Signaling Technology	Cat# 18640S
Rabbit monoclonal anti-FLIP	Cell Signaling Technology	Cat# 56343
Mouse monoclonal anti-FADD	Adipogen Life Sciences	Cat# AG-20B-0080-C100
Rabbit polyclonal anti-ZDHHC5	Biodragon	Cat# BD-PN6038
Rabbit polyclonal anti-ZDHHC9	Biodragon	Cat# BD-PT1344
Rabbit polyclonal anti-ZDHHC12	Biodragon	Cat# BD-PN3933
Rabbit polyclonal anti-ZDHHC17	Abclonal	Cat# A6793
Rabbit polyclonal anti-ZDHHC20	Abclonal	Cat# A17982
Mouse monoclonal anti-HPIV3 HN	Abcam	Cat# ab252769
Mouse monoclonal anti-RSV phosphoprotein (P)	Abcam	Cat# ab94965
Rabbit monoclonal anti-RSV nucleoprotein (N)	GeneTex	Cat# GTX636711
Mouse monoclonal anti-GAPDH	ABclonal	Cat# AC033
Rabbit monoclonal anti-β-actin	ABclonal	Cat# AC026
Goat anti-Mouse IgG (H+L) Secondary Antibody, HRP	Thermo Fisher Scientific	Cat# 31430
Goat anti-Rabbit IgG (H+L) Secondary Antibody, HRP	Thermo Fisher Scientific	Cat# 31460
Alexa Fluor™ 488 goat anti-mouse IgG (H + L) secondary antibody	Thermo Fisher Scientific	Cat# A-11029
Alexa Fluor™ 594 goat anti-rabbit IgG (H + L) secondary antibody	Thermo Fisher Scientific	Cat# A-11012

Table S3. Reagents

Reagants	Source	Identifier
Phorbol 12-myristate 13-acetate (PMA)	Sigma-Aldrich	Cat# P1585
Lipopolysaccharides	Sigma-Aldrich	Cat# L-2880
Nigericin	Invivogen	Cat# tlrl-nig
Poly(dA:dT) naked	Invivogen	Cat# tlrl-patn
Talabostat mesylate (Val-boroPro mesylate)	MCE	Cat# HY-13233A
Cycloheximide	MCE	Cat# HY-12320
Recombinant human TNF-α (carrier-free)	Biolegend	Cat# 570106
5z-7-Oxozeaenol	MCE	Cat# HY-12686
Fas ligand protein (FasL), human	MCE	Cat# HY-P72658
Birinapant（TL32711）	ApexBio	Cat# A4219
SM-164	MCE	Cat# HY-15989
Navitoclax	MCE	Cat# HY-10087
Cisplatin	Sigma-Aldrich	Cat# 479306
MCC950 sodium	MCE	Cat# HY-12815A
Z-VAD-FMK	MCE	Cat# HY-16658B
Belnacasan (VX-765)	Selleck	Cat# S2228
Z-LEHD-FMK	MCE	Cat# HY-P1010
Z-AEVD-FMK	APExBIO	Cat# C3322
Wortmannin	MCE	Cat# HY-10197
LY294002	MCE	Cat# HY-10108
Bortezomib	MCE	Cat# HY-10227
3-Methyladenine (3-MA)	Selleck	Cat# S2767
Chloroquine (CQ)	MCE	Cat# HY-17589A
MG-132	MCE	Cat# HY-13259
Dimethyl sulfoxide (DMSO)	Sigma-Aldrich	Cat# D2650
Disuccinimidylsuberate (DSS)	Sigma-Aldrich	Cat# S1885
Polybreneρ	Santa Cruz Biotechnology	Cat# sc-134220
Puromycin	Sigma-Aldrich	Cat# 540411
Blasticidin S	YESEN	Cat# 60218ES10
NEOFECT™ DNA transfection reagent	Neofect biotech	Cat# TF201201
Lipofectamine™ LTX reagent with PLUS™ reagent	Thermo Fisher Scientific	Cat# 15338030
Protease inhibitor cocktail	MCE	Cat# HY-K0010
Phenylmethylsulfonyl fluoride (PMSF)	Sigma-Aldrich	Cat# 329-98-6
SYTOX green nucleic acid stain	Thermo Fisher Scientific	Cat# S7020
DAPI	Sigma-Aldrich	Cat# D9542
ProLong™ Diamond Antifade Mountant	Thermo Fisher Scientific	Cat# P36970
Hoechst 33342 staining solution for live cells, 100 ×	Beyotime	Cat# C1029
Paraformaldehyde (PFA)	Sigma-Aldrich	Cat# P6148
Trizol	Thermo Fisher Scientific	Cat# 15596026CN
PEG6000	BBI LIFE SCIENCES	Cat# A610432-0500
Triton X-100	BioFroxx	Cat# 1139ML100
Tween 20	BioFroxx	Cat# 1247ML100
BSA	BioFroxx	Cat# 4240GR500
EDTA	BioFroxx	Cat# 1340GR100

**Continued**		
**Reagants**	**Source**	**Identifier**
SDS	BioFroxx	Cat# 3250KG001
DTT	BioFroxx	Cat# 1111GR005
Modified Bradford reagent	BBI life sciences	Cat# C100530-0100
Clarity Western ECL Substrate	Bio-Rad	Cat# 1705061
